# Supplementary material for: Distinct Structural Pathways Coordinate the Activation of AMPA Receptor-Auxiliary Subunit Complexes
Source: Neuron. 2016 Mar 16;89(6):1264–76. doi: 10.1016/j.neuron.2016.01.038 (PMC4819453; doi:10.1016/j.neuron.2016.01.038)
Supplement: Document S1. Supplemental Experimental Procedures, Figures S1–S7, and Tables S1–S3 [file mmc1.pdf]

Neuron, Volume *89*

Supplemental Information

Distinct Structural Pathways Coordinate the Activation of AMPA Receptor-Auxiliary Subunit Complexes

G. Brent Dawe, Maria Musgaard, Mark R.P. Aurousseau, Naushaba Nayeem, Tim Green, Philip C. Biggin, and Derek Bowie

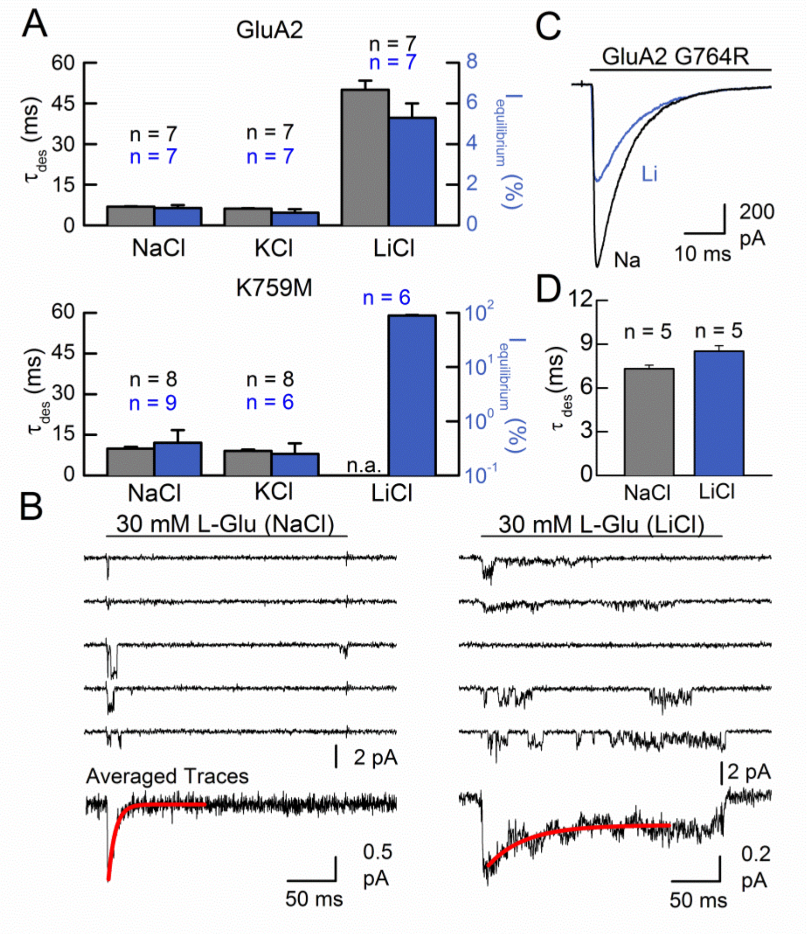

Figure S1 Functional properties of GluA2 receptors in different external ionic conditions. Supplemental data associated with Figure 1.

Since cation substitution experiments at GluA1 AMPARs do not affect the time course of channel activation (Bowie, 2002), we have explored whether GluA2 AMPARs are sensitive to other cation species beside lithium (i.e. potassium), and whether any structural features account for the difference in ion modulation between AMPAR subunits. One difference between GluA1 and GluA2 AMPARs is the R/G RNA editing site, located at the apex of the LBD, adjacent to the electronegative pocket on the same and opposing (across the dimer interface) subunits. This residue is edited (i.e. Gly) in GluA2, but unedited (i.e. Arg) in GluA1 (Lomeli et al., 1994). To determine whether the editing state of the R/G site affects lithium modulation of GluA2, we measured desensitization kinetics in the GluA2 G764R mutant.

(A) Current decay time constants (left, grey) and equilibrium to peak current ratios (right, blue) for wildtype GluA2 (top) and K759M (bottom) mutant receptors in the presence of different

cation species. Data are mean ± SEM, from the number of independent patch experiments indicated.

(B) Typical GluA2 unitary current events elicited by 30 mM L-Glu in NaCl (left) (Patch #140128p7) and LiCl (right) (Patch # 140121p10) external solutions. Below is an average of several individual sweeps, including a single, exponential function fit (red) of the current decay.

(C) Typical current responses elicited by 10 mM L-Glu on GluA2 G764R mutant receptors in external NaCl and LiCl (Patch # 150203p3).

(D) Average current decay time constants for GluA2 G764R receptors. Consistent with the behavior of GluA1 receptors, the slowing of wildtype GluA2 receptor decay kinetics observed with lithium was almost entirely eliminated in G764R mutant receptors, which exhibited desensitization time constants of 7.3 ± 0.2 ms (n = 5) in NaCl and 8.5 ± 0.4 ms (n = 5) in LiCl. Data are mean ± SEM, from the number of independent patch experiments indicated.

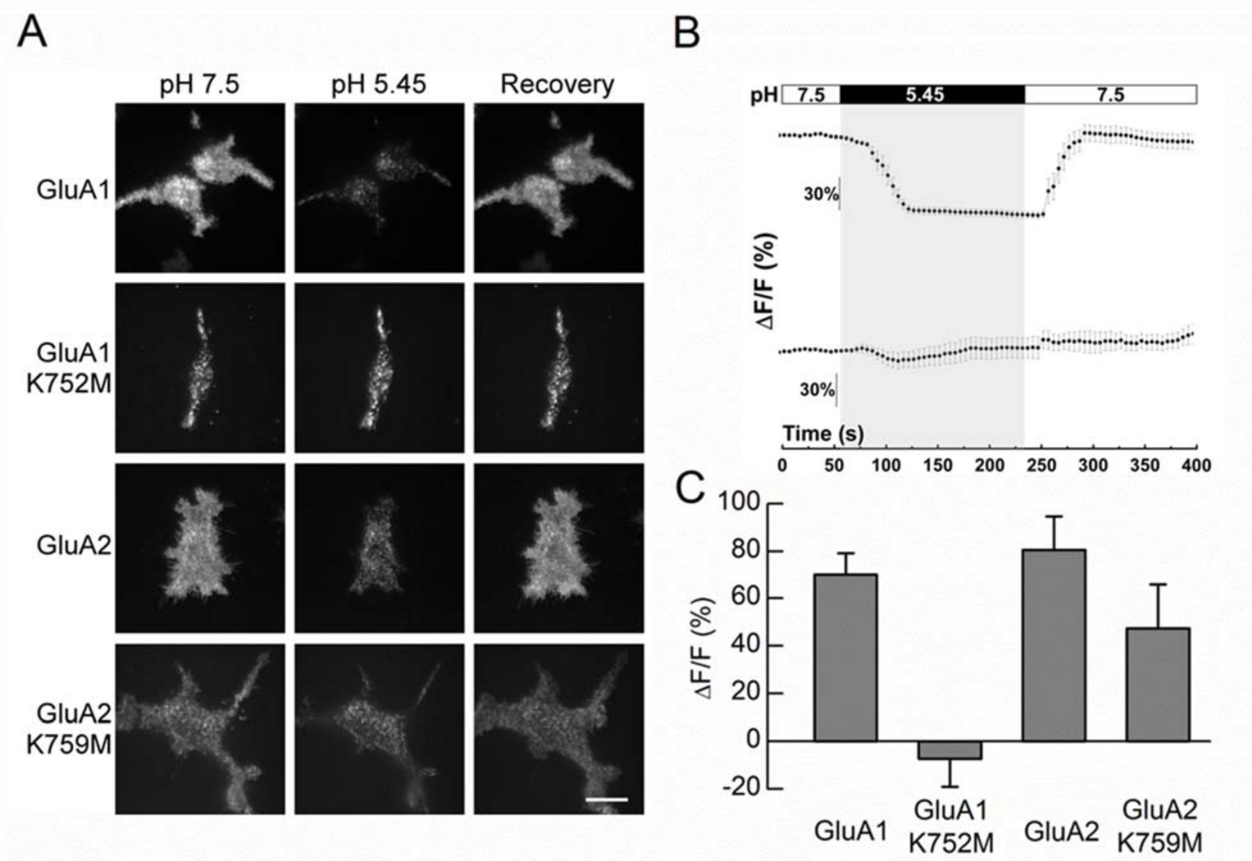

Figure S2 Surface expression of AMPAR subunits harboring an equivalent Lys to Met mutation at the electronegative pocket. Supplemental data associated with Figure 2.

(A) TIRF images of HEK293T cells transfected with GFP-tagged wildtype and mutant GluA1 and GluA2 receptors exhibit reversible attenuation of the fluorescence signal between pH 7.5 and 5.45 when subunits are expressed on the plasma membrane (scale bar = 20 µm).

(B) Individual, time-resolved fluorescence profiles for single cells expressing either wildtype GluA1 (top) or the K752M mutant (bottom).

(C) Bar graph tabulating the change in fluorescent signal observed for cells expressing wildtype and mutant AMPARs. Data are mean ± SEM, from five (GluA1 K752M), ten (wildtype GluA1), thirteen (GluA2 K759M), or eighteen (wildtype GluA2) individual cell imaging experiments for each receptor.

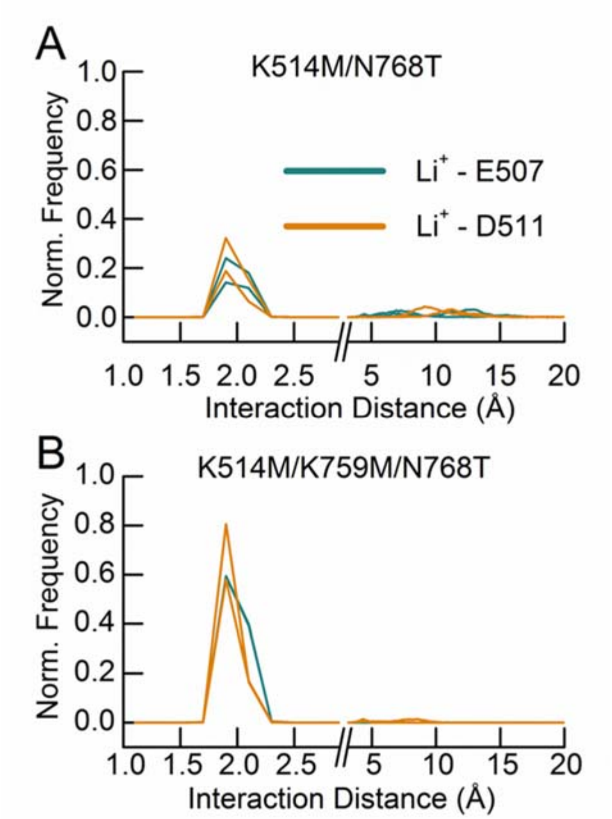

Figure S3 Lithium binding properties of the GluA2 K514M/N768T mutant receptor. Supplemental data associated with Figure 3.

(A and B) Data from MD simulations reporting the interaction distance between residue Glu507 or Asp511and the nearest lithium ion, for K514M/N768T (A) and K514M/K759M/N768T (B) mutant GluA2 receptors. Data from the triple mutant (i.e. K514M/N768T + K759M) was included to take advantage of the increased frequency of lithium binding measured in prior simulations following addition of the K759M mutation (Figure 1). Distance was measured from the sidechain oxygen atom closest to lithium on the residues indicated. Frequency is normalized (bin size = 0.2 Å, cumulative frequency = 1.0) and averaged from two simulation repeats of 100 ns for each receptor. Values for each chain (A and B) in the LBD dimer are shown.

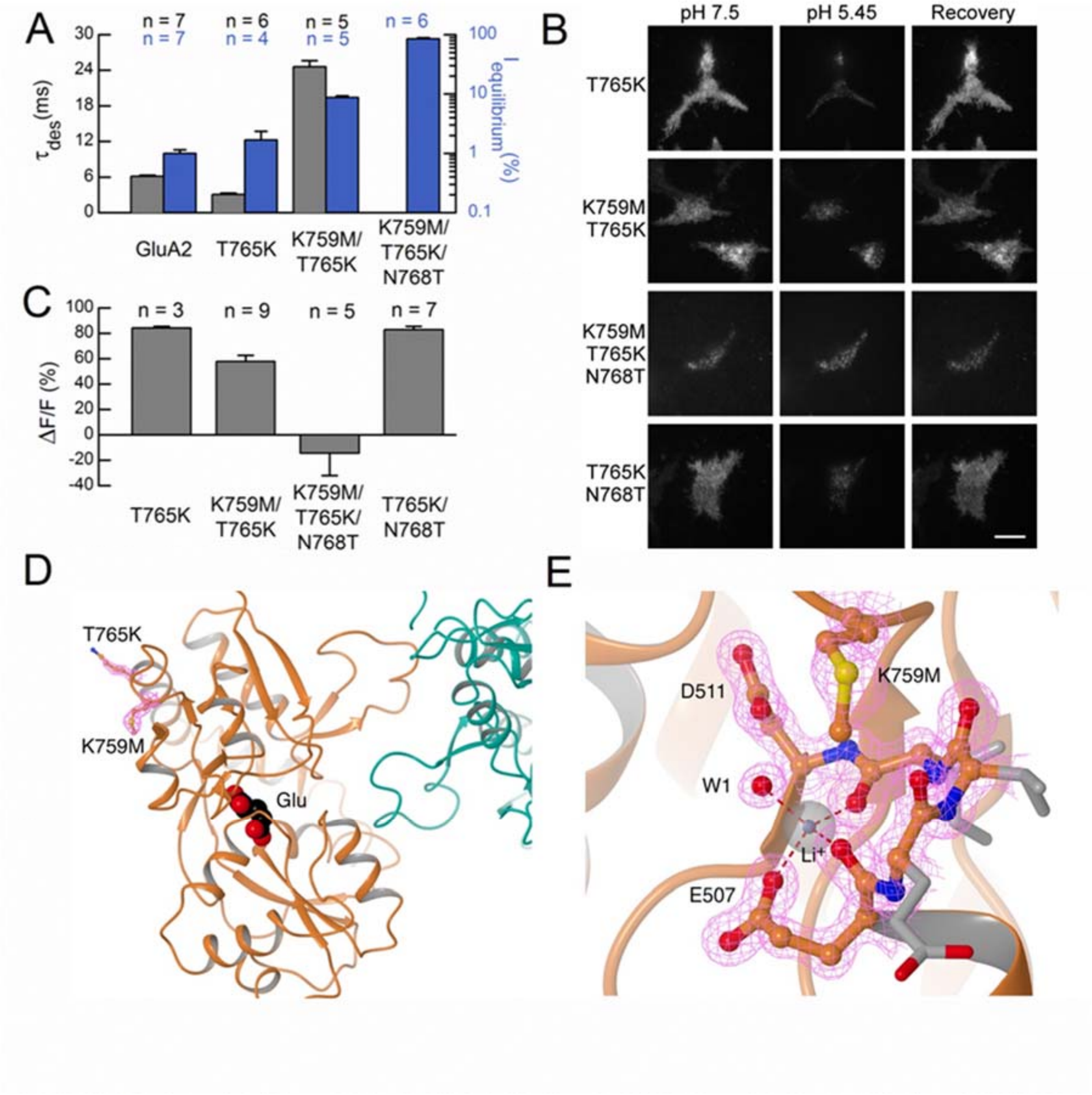

Figure S4 Structure, function, and surface expression of the GluA2 T765K mutant series of receptors. Supplemental data associated with Figure 4.

Our previous work has established that GluK2 KAR desensitization is abolished by the D776K mutation, which acts as a cross-dimer electrostatic tether onto the cation binding pocket (Dawe et al., 2013; Nayeem et al., 2011; Nayeem et al., 2009). We created a series of mutants incorporating the equivalent mutation in GluA2, namely T765K, in the hopes of achieving a similar tether between GluA2 subunits. Perhaps because Lys759 interferes with the tethering of the mutant lysine at the electronegative pocket of GluA2 (Figure 1), the addition of the K759M mutation was required atop T765K to prolong the time course of current responses. As such, we focussed our analysis on GluA2 K759M/T765K, for which we were able to crystalize a

crosslinked LBD dimer, and also GluA2 K759M/T765K/N768T (MKT), which showed little, if any, detectable current decay over 250 ms L-Glu applications. Nevertheless, kinetic analysis of GluA2 MKT was made difficult due to its greatly diminished surface expression.

(A) Time constants of current decay (left, grey) and equilibrium to peak current ratio (right, blue) for the GluA2 T765K series of mutants. Data are mean ± SEM, from the number of independent patch experiments indicated.

(B) TIRF images of HEK293T cells transfected with wildtype GluA2 or one of several mutant receptors possessing Lys at the 765 position. Reversible attenuation of the GFP fluorescence signal occurs between pH 7.5 and 5.45 when subunits are expressed on the plasma membrane (scale bar = 20 μm).

(C) Bar graph tabulating the change in fluorescent signal observed for cells expressing wildtype and mutant AMPARs. Data are mean ± SEM, from the number of independent patch experiments indicated.

(D) View of protomer A (orange), with the two mutated residues shown with associated electron density ($|2F_{obs} - F_{calc}|\alpha_{calc}$; pink mesh, contoured at 1.5σ). The L-Glu ligand is shown in black space-fill. Part of protomer B (teal) can be seen to the right, highlighting the absence of the biological dimer from this crystal form.

(E) Closer view of protomer A (orange) from approximately the viewpoint shown in Figure 4. The modeled lithium ion (gray sphere) is shown, along with an interacting water (W1) and other atoms within the electronegative pocket. Electron density ($|2F_{obs} - F_{calc}|\alpha_{calc}$) is shown contoured at 2σ around the displayed atoms (pink mesh) with the exception of the lithium ion, where it is displayed at 1σ (gray mesh). Contacts between the lithium ion and other atoms are shown as dashed lines.

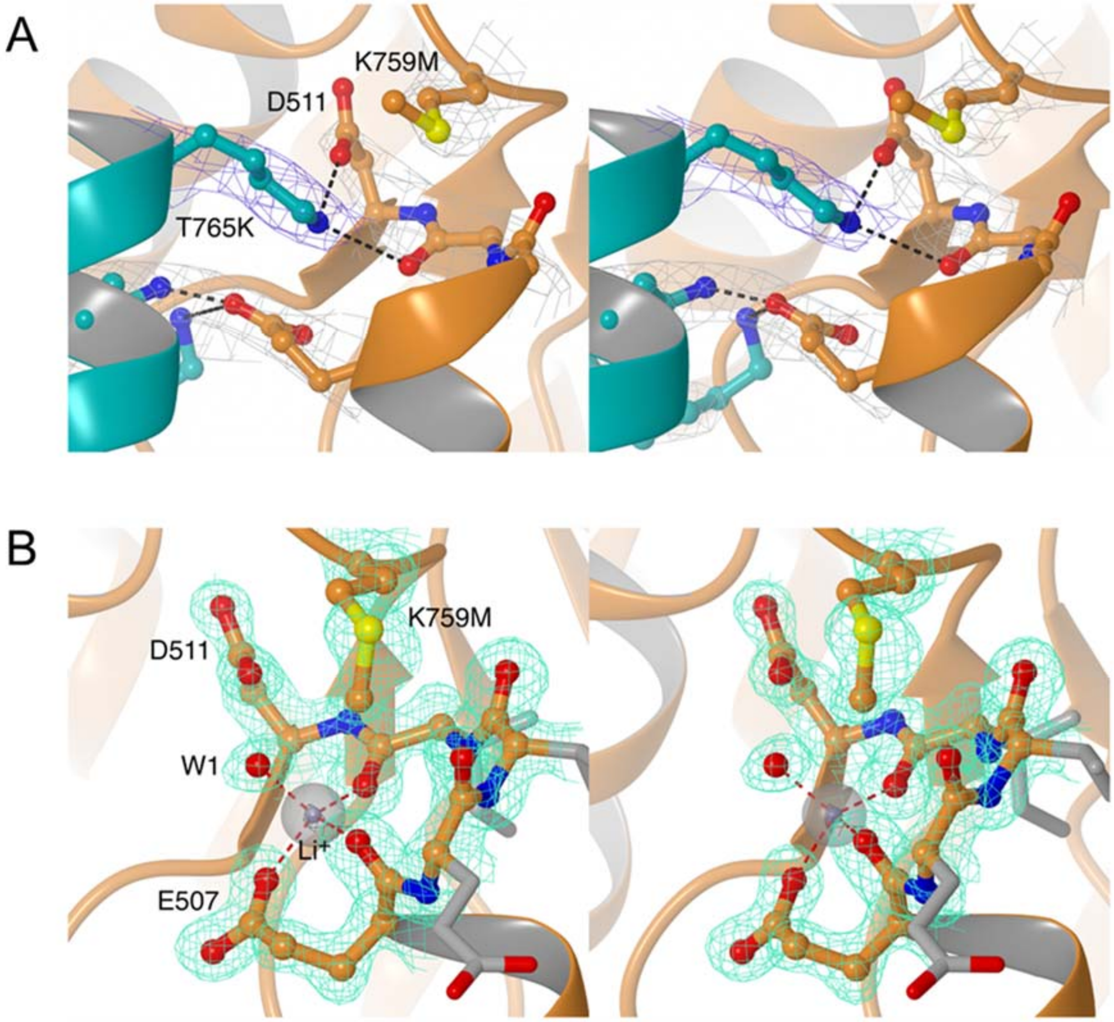

Figure S5 Stereo views of composite omit maps of the GluA2 K759M/T765K LBD, generated using PHENIX. Supplemental data associated with Figure 4.

(A) View of the mutant T765K residue on protomer B of the zinc crystal form, interacting with residues in the electronegative pocket of protomer A. Residue and density labelling is maintained as for Figure 4, with contours displayed at 1.2 σ.

(B) View of the electronegative binding pocket on protomer A of the lithium crystal form. Residue and density labelling is maintained as for Supplemental Figure 4, with contours displayed at 1.5 σ (or 0.8 σ around the lithium ion).

			Helix D		Helix J
NP_113796.1	GluA1	*494*	TITLVREEVIDFSKP	*754*	SALRNPVNLAVLKL
NP_058957.1	GluA2	*501*	TITLVREEVIDFSKP	*761*	SSLGTPVNLAVLKL
NP_116785.2	GluA3	*504*	TITLVREEVIDFSKP	*766*	SALGTPVNLAVLKL
NP_058959.2	GluA4	*502*	TITLVREEVIDFSKP	*762*	SSLRTPVNLAVLKL
NP_058937.1	GluK1	*533*	TITYVREKVIDFSKP	*787*	SPYRDKITIAILQL
NP_062182.1	GluK2	*518*	AITYVREKVIDFSKP	*772*	SPYRDKITIAILQL
NP_852038.2	GluK3	*520*	TITHVREKAIDFSKP	*773*	SPYRDKITIAILQL
NP_036704.1	GluK4	*502*	TITAEREKVIDFSKP	*757*	SVFRDEFDLAILQL
NP_113696.1	GluK5	*501*	TITAEREKVIDFSKP	*756*	SPFRDEITLAILQL
NP_058706.1	GluN1	*518*	TINNERAQYIEFSKP	*766*	SPWKQNVSLSILKS
NP_036705.3	GluN2A	*513*	TINEERSEVVDFSVP	*769*	SPWKRQIDLALLQF
NP_036706.1	GluN2B	*514*	TINEERSEVVDFSVP	*770*	SGWKRQVDLAILQL
NP_036707.3	GluN2C	*524*	TINEERSEIIDFSVP	*780*	SHWKRAIDLALLQL
NP_073634.1	GluN2D	*538*	TINEERSEIVDFSVP	*794*	SRWKRPIDLALLQF
NP_612555.1	GluN3A	*633*	SINTARSQVIDFTSP	*881*	SPLTSNISELISQY
NP_579842.2	GluN3B	*524*	SINSARSQVVDFTSP	*781*	SPLTSNLSEFISRY

Figure S6 Amino acid sequence alignment of iGluRs at the apical LBD dimer interface. Supplemental data associated with Figure 5.

Amino acid sequences of iGluR subunits from *R. norvegicus*, aligned over two segments of the LBD. The NCBI accession code is shown at left. For AMPAR subunits, the flip isoform was selected. Residues participating in cross-dimer electrostatic interactions at the apex of the GluA2 LBD are highlighted magenta. When these residues are not conserved with the equivalent AMPAR residue they are highlighted cyan. The conserved Phe residue at position 512 could not be mutated alongside other resides in the GluA2 AAA mutant, since its contribution to the electrostatic network is from a backbone oxygen atom. Nevertheless, both residues across the dimer interface that would be expected to interact with Phe512, namely Lys514 and Asn768, were truncated.

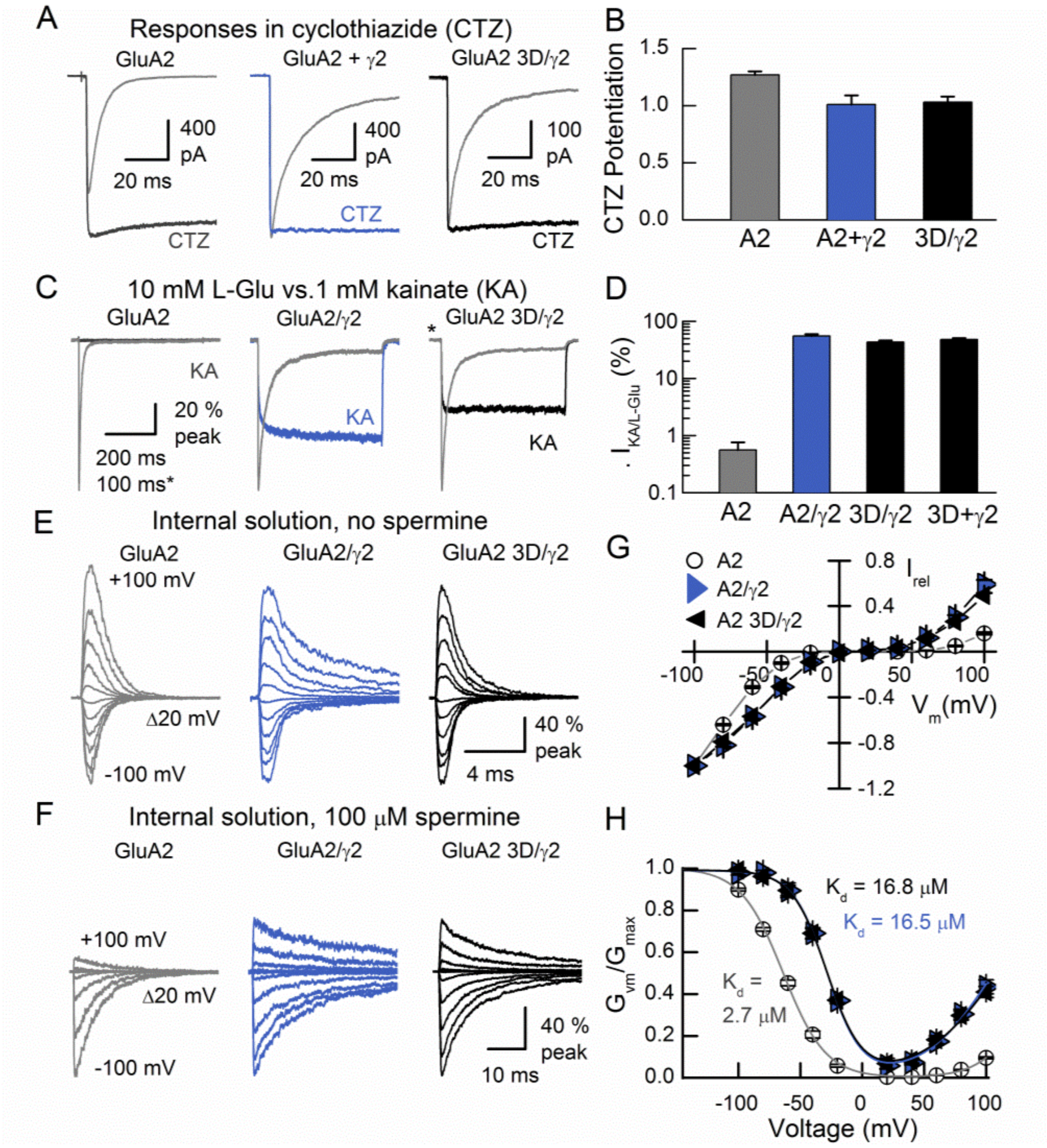

Figure S7 The GluA2 3D mutation does not attenuate TARP γ2 modulation of apparent agonist efficacy or channel block by spermine. Supplemental data associated with Figure 7.

(A) Typical current responses to 10 mM L-Glu before (grey) and during CTZ exposure for wildtype GluA2 (Patch # 130217p8, dark grey), as well as co-expressed GluA2 + γ2 (Patch # 150305p7, blue) and the GluA2 3D/γ2 (Patch # 150911p1, black) AMPAR-TARP fusion protein.

(B) Mean CTZ potentiation of the receptors described in panel A. Values for GluA2 and GluA2 + γ2 correspond to those reported in Figures 5 and 6. Data are mean ± SEM, from eleven (GluA2), five (GluA2 + γ2), or six (GluA2 3D/γ2) independent patch experiments.

(C) Scaled current responses to 10 mM L-Glu (grey) and 1 mM KA for wildtype GluA2 (Patch # 150317p3, dark grey), as well as GluA2/γ2 (Patch # 150316p10, blue) and GluA2 3D/γ2 (Patch # 150511p6, black) AMPAR-TARP fusion proteins.

(D) Mean 1mM KA response, as a percentage of the peak current yielded by 10 mM L-Glu, for the receptors in panel C, as well as GluA2 3D + γ2 (TARP co-expressed). Data are mean ± SEM, from five (GluA2 and GluA2 3D/γ2), six (GluA2/γ2) and seven (GluA2 3D + γ2) independent patch experiments.

(E) Scaled current responses to 10 mM L-Glu at a range of membrane potentials (-100 to +100 mV, Δ20 mV) for wildtype GluA2 (Patch # 150716p10, grey), as well as GluA2/γ2 (Patch # 150723p11, blue) and GluA2 3D/γ2 (Patch # 150911p6, black) AMPAR-TARP fusion proteins.

(F) Scaled current responses to 10 mM L-Glu at a range of membrane potentials (-100 to +100 mV, Δ20 mV) with 100 μM spermine added to the internal recording solution for wildtype GluA2 (Patch # 150525p5, grey), as well as GluA2/γ2 (Patch # 150528p11, blue) and GluA2 3D/γ2 (Patch # 150910p7, black) AMPAR-TARP fusion proteins.

(G) Current-voltage plots with 100 μM internal spermine for wildtype GluA2 (white circles), as well as GluA2/γ2 (blue triangles) and GluA2 3D/γ2 (black triangles) AMPAR-TARP fusion proteins. Currents were normalized to the response at -100 mV (I_{rel} = -1). Data are mean ± SEM, from six (GluA2, GluA2/γ2, and GluA2 3D/γ2) independent patch experiments.

(H) Conductance-voltage plots with 100 μM internal spermine for wildtype GluA2 (white circles), as well as GluA2/γ2 (blue triangles) and GluA2 3D/γ2 (black triangles) AMPAR-TARP fusion proteins. Conductance is normalized to the fitted maximal conductance (G_{max}), and corrected to account for the average conductance-voltage relationship in recordings without internal spermine (see Supplemental Experimental Procedures). Data are mean ± SEM, from six independent patch experiments for each condition (GluA2, GluA2/γ2, and GluA2 3D/γ2) with 100μM spermine.

Table S1 Data collection and refinement statistics (molecular replacement) for GluA2 K759M/T765K LBD crystal structures. Supplemental data associated with Figure 4.

Data set	GluA2 K759M/T765K zinc-form	GluA2 K759M/T765K lithium-form
**Data collection**		
Space group	$P\ 2\ 2_1\ 2_1$	P 1 2 1
Cell dimensions		
a, *b*, *c* (Å)	46.38, 110.52, 167.26	67.32, 47.56, 96.75
α, β, γ (°)	90, 90, 90	90, 95.65, 90
Resolution (Å)	2.90-92.2 (2.90-3.08) [a]	1.35-67.0 (1.35-1.39)
R_{meas}	0.225 (1.68)	0.074 (1.11)
$I / \sigma I$	4.5 (1.1)	10.92 (1.88)
CC(1/2)	98.8 (73.9)	99.7 (62.2)
Completeness (%)	100 (100)	94.7 (92.5)
Redundancy	5.0 (5.1)	3.5 (3.6)
**Refinement**		
Resolution (Å)	2.90	1.35
No. reflections	36,525	126,864
R_{work} / R_{free} [b]	0.243 / 0.283	0.161 / 0.178
No. atoms		
Protein	5873	4143
Ligands (Glu only)	30 (30)	52 (20)
Ions	5	2
Water	0	688
B-factors		
Protein	97.1	23.2
Ligands (Glu only)	89.7 (89.7)	33.9 (15.4)
Ions	119.4	11.2
R.m.s. deviations		
Bond lengths (Å)	0.006	0.011
Bond angles (°)	1.035	1.391

Data were collected from single crystals in each case.

[a] Values in parentheses are for highest-resolution shell.

[b] Tests sets for R_{free} contained 5% (zinc-form) or 2% (lithium-form) of total reflections.

Table S2 Time course of desensitization and deactivation for wildtype and mutant GluA2 receptors expressed alone, with auxiliary subunits, or as GluA2/γ2 fusion proteins. Supplemental data associated with Figures 6, 7, and 8.

	τ_{fast}	τ_{slow}	% fast	$\tau_{weighted}$	$\tau_{monoexponential}$	n
AMPAR subunits						
GluA2						
desensitization	7.9 ± 0.7	36.8 ± 8.3	94 ± 1	**9.2 ± 0.8**	**8.7 ± 0.71**	9
deactivation	0.61 ± 0.07	6.2 ± 0.9	96 ± 1	**0.76 ± 0.08**	**0.67 ± 0.07**	8
3D mutant series						
GluA2/y2						
desensitization	22.4 ± 2.4	133.0 ± 17.3	79 ± 3	**45.7 ± 6.8**		11
deactivation	1.4 ± 0.3	13.8 ± 2.2	84 ± 3	**3.2 ± 0.4**		9
GluA2 3D/y2						
desensitization	7.9 ± 0.9	32.3 ± 3.1	80 ± 3	**12.7 ± 1.2**		8
deactivation	0.83 ± 0.05	6.6 ± 1.2	93 ± 2	**1.1 ± 0.1**		8
GluA2 3D + γ2						
desensitization	5.8 ± 0.6	25.5 ± 3.7	76 ± 6	**9.5 ± 0.4**		7
deactivation	0.69 ± 0.05	16.4 ± 3.3	100 ± 0	**0.74 ± 0.04**	**0.67 ± 0.07**	7
AAA mutant series						
GluA2 + γ2						
desensitization	20.5 ± 3.6	86.1 ± 15.8	65 ± 7	**39.0 ± 4.4**		10
GluA2 + γ7						
desensitization	9.8 ± 1.0	58.7 ± 8.3	87 ± 1	**16.0 ± 1.5**		7
GluA2 AAA + γ2						
desensitization	2.5 ± 0.3	16.9 ± 1.4	73 ± 4	**6.6 ± 0.9**		8
GluA2 AAA + γ7						
desensitization	0.91 ± 0.10	27.0 ± 8.5	99 ± 0	**1.1 ± 0.1**	**0.92 ± 0.10**	6
GluA2 AAA/3D + γ2						
desensitization	1.1 ± 0.1	14.6 ± 2.3	89 ± 2	**2.4 ± 0.3**		7

GluA2 receptors were activated by long application (250 or 500 ms) or short (1 ms) applications of 10 mM glutamate to measure desensitization and deactivation kinetics, respectively. In the presence of auxiliary subunits, current decay associated with desensitization and deactivation was fit using bi-exponential functions to obtain the components τ_{fast} and τ_{slow}. Weighted time constants ($\tau_{weighted}$) were calculated based on the relative area fit by the fast and slow components. In cases where the τ_{fast} accounted for 94 % or more of the total area, the decay was instead fit by a monoexponential function, as reported as the value in the $\tau_{monoexponential}$ column. The number of patches for each condition (n) is indicated, and all values are mean ± SEM.

Table S3 Spermine affinities of GluA2 receptors. Supplemental data associated with Figure 7.

Fit parameters of GluA2 G/V relationships

Receptor		Mean	SEM	n
GluA2	Kd (0 mV) (μM)	2.7	0.4	
100 μM spermine	h (mV)	-17.3	0.6	6
	k (mV)	16.5	0.2	
0 μM spermine	G_0	1.09		
	V	53.2		
GluA2/γ2	Kd (0 mV) (μM)	16.5	1.3	
100 μM spermine	h (mV)	-14.0	1.0	6
	k (mV)	21.5	0.9	
0 μM spermine	G_0	1.14		
	V	82.4		
GluA2 3D/γ2	Kd (0 mV) (μM)	16.8	2.1	
100 μM spermine	h (mV)	-14.2	1.1	6
	k (mV)	25.2	0.6	
0 μM spermine	G_0	1.09		
	V	74.0		

Affinities were obtained using responses evoked by 250 or 500 ms applications of 10 mM L-Glu. Values for 'h' and 'k' indicate the voltage dependency. The number of patches for each condition (n) is indicated, and all values are mean ± SEM.

Movie S1A Lithium occupancy of the putative cation binding pocket of GluA2 K759M. Supplemental movie associated with Figure 1.

MD simulation of the GluA2 K759M LBD dimer shows that lithium (magenta) stably occupies the electronegative pocket throughout the simulation. Additional details regarding simulation parameters are described in the online methods section and quantification of the MD results are shown in Figure 1. The video encompasses 100 ns of the simulation.

Movie S1B Sodium does not occupy of the putative cation binding pocket of GluA2 K759M. Supplemental movie associated with Figure 1.

MD simulation of the GluA2 K759M LBD dimer shows that sodium (magenta) only rarely comes into contact with the electronegative pocket during the simulation. Additional details regarding simulation parameters are described in the online methods section and quantification of the MD results are shown in Figure 1. The video encompasses 100 ns of the simulation.

Movie S4A Rupture of T765K tether onto the cation pocket residues of GluA2 K759M/T765K. Supplemental movie associated with Figure 4.

MD simulation of the GluA2 K759M/T765K LBD dimer shows T765K initially maintaining contact with the electronegative residues that form the pocket, but then losing this interaction in the latter half of the simulation. Additional details regarding simulation parameters are described in the online methods section and quantification of the MD results are shown in Figure 4. The video encompasses 100 ns of the simulation.

Movie S4B Unstable tethering of T765K onto the cation pocket residues of GluA2 MKT. Supplemental movie associated with Figure 4.

MD simulation of the GluA2 MKT dimer shows T765K initially maintaining contact with the electronegative residues that form the pocket, but then having difficulty preserving this interaction in the latter half of the simulation. Additional details regarding simulation parameters are described in the online methods section and quantification of the MD results are shown in Figure 4. The video encompasses 100 ns of the simulation.

SUPPLEMENTAL EXPERIMENTAL PROCEDURES

DNA constructs

The GluA2/γ2 and GluK2/γ2 TARP fusion constructs were generated by large-insert site-directed mutagenesis (see (Geiser et al., 2001)). The γ2 coding sequence was amplified as part of a megaprimer, and then subsequently incorporated into plasmids encoding either the GluA2 or GluK2 iGluR subunit. The forward primer used to amplify the megaprimer corresponded to the C-terminal of the GluA2 or GluK2 (without its stop codon), a seven amino acid linker sequence ELGTRGS (Semenov et al., 2012), and the N-terminal of γ2. Likewise, the reverse primer corresponded to a region downstream of the iGluR subunit coding region and the C-terminal of γ2. The primer sequences used to generate the megaprimer for the GluA2/γ2 fusion protein were 5'- GGC ATC GAG AGT GTT AAA ATT GAA CTG GGT ACA CGA GGT TCT ATG GGG CTG TTT GAT CGA GGT G -3' (forward primer) and 5'- GTA ATT GAC AGC CTT GCC TTG CTC CTC ATT TCT CAT ACG GGC GTG GTC CG -3' (reverse primer), while for the GluK2/γ2 fusion protein they were 5'- CCA GGT AAA GAA ACT ATG GCA GAA CTG GGT ACA CGA GGT TCT ATG GGG CTG TTT GAT CGA GGT G -3' (forward primer) and 5'- CGA CAG TTT GTG CTT GGG TGA TTG GCC TCT TCT CAT ACG GGC GTG GTC CG -3' (reverse primer). All new constructs were screened by restriction digestion and confirmed by sequencing.

Electrophysiology

cDNA encoding AMPAR or KAR subunits and green fluorescent protein (eGFP) was typically co-transfected, but in some cases receptor subunit cDNA was present on plasmids also encoding eGFP behind an internal ribosomal entry site. For GluA1 and GluA2 AMPARs, the Q/R site was unedited, while the R/G site was unedited and edited, respectively. After transfection for 4 - 16

hours using the calcium phosphate precipitation method, cells were washed twice with divalent-containing PBS and maintained in fresh medium (MEM containing Glutamax and 10 % FBS), including 30 μM DNQX if auxiliary subunits were present. Electrophysiological recordings were performed 24 - 48 hours later. The osmotic pressure of recording solutions was adjusted to 300 mOsm using sucrose, while the pH was typically adjusted to 7.4 with alkali hydroxide solutions. For experiments involving spermine in the patch pipette the internal solution contained (in mM): 120 NaCl, 10 NaF, 5 HEPES, 5 Na_4BAPTA, and 0.5 $CaCl_2$ with 100 μM spermine added on the day of experiments. In the case of recordings conducted without external NaCl, the solution contained 100 μM of $CaCl_2$ and $MgCl_2$ to improve patch stability, sucrose to maintain the osmotic pressure at 300 mOsm, and 5 mM Tris to buffer pH. The pH was further adjusted to 7.4 using 10 N HCl. In the case of single-channel recordings, internal solution typically contained (in mM): 135 CsF, 33 CsOH, 10 HEPES, 11 EGTA, 1 $CaCl_2$, and 2 $MgCl_2$.

All experiments were performed on excised membrane patches in the outside-out configuration. Recording pipettes were composed of borosilicate glass (3-5 MΩ, King Precision Glass) coated with dental wax, or quartz glass (3-15 MΩ, King Precision Glass) coated with Sylgard (Dow Corning) to obtain recordings of single channels or for stable recordings in external solution without NaCl. The reference electrode was connected to the bath via an agar bridge of 3M KCl. The holding potential during recordings was -60 mV (unless otherwise stated). Series resistances (3-15 MΩ) were routinely compensated by 95 %. For single-channel recordings, the headstage was set to the capacitive feedback recording mode. All recordings were performed using an Axopatch 200B amplifier (Molecular Devices). Current records were low-pass filtered by an 8-pole Bessel filter at 10 kHz and sampled at 25-100 kHz for population responses or 100 kHz for

single-channel responses. Data were acquired using pClamp9 software (Molecular Devices) and illustrated using Origin 7 (OriginLab).

Analysis of Electrophysiological Data

Electrophysiological data containing population and single-channel responses were analyzed using Clampfit 9.0 (Molecular Devices) and Signal 5.0 (Cambridge Electronic Design), respectively. Current decay rates were fit using 1st or 2nd order exponential functions: $y = A_i * \exp(-x/t_i)$, with the latter used when auxiliary subunits were present (see Table S2). Single-channel data were processed as described previously (Dawe et al., 2013). In brief, digital low-pass filtering at 3 kHz was performed prior to time-course fitting, which resulted in root mean square baseline noise values that averaged 0.22 ± 0.02 pA (n = 4) and 0.18 ± 0.01 pA (n = 4) for wildtype GluA2 and MKT mutant receptors, respectively. These noise values corresponded to approximately fifty percent of the smallest conductance level. Idealized record response amplitudes were fit with Gaussian functions, whose peaks reflect discrete conductance levels: $y = \sum_{i=1...n} (A_i/(w_i*\mathrm{sqrt}(\pi/2)))*\exp(-2*((x-xc_i)/w_i)^2)$ where A = area, xc = center of the peak, w = error associated with xc. Open probability was calculated for each patch containing GluA2 MKT as the percentage of open time in the idealized record.

Fitting of Conductance Voltage Relationships

Agonist-evoked membrane conductance (G) was calculated using the equation: $G = I / (V - V_{rev})$, where I is the current at V holding potential, and V_{rev} is the reversal potential. Conductance-voltage (G/V) relationships were fit using Origin 7 (OriginLab) with two different equations (Bowie et al., 1998). For recordings without internal polyamines, $y = (1 + (G_0 - 1)*\exp(x/V))$ was used, where G_0 is the minimal conductance and V is the holding potential. For recordings with internal polyamines, $y = G_{max} / (1 + [PA]/(g*\exp(x/h) + L*\exp(x/k)))$ was used,

where G_{max} is the maximal conductance and [PA] is the concentration of polyamine (in μM), such that the polyamine dissociation constant, K_d = g*exp(V/h) + L*exp(V/k) (see (Bowie et al., 1998)). For each receptor studied, the K_d(0 mV) and the voltage-dependent rates h an k are reported (Table S3). Conductance-voltage data from patch recordings with internal polyamines were corrected based on the average conductance profile of the same receptor without polyamines. In some cases, residual polyamine block was detected during outside-out patch recordings, despite the presence of 10 mM ATP to chelate polyamines in the patch pipette. To eliminate this block during control experiments, a train of L-Glu pulses at -80 mV were delivered prior to the test pulse, as described previously (Rozov et al., 1998).

Molecular dynamics simulations

The L-Glu-bound GluA2 (flip, R/G unedited) LBD structure (PDB # 2UXA (Greger et al., 2006); resolution 2.38 Å) was obtained from the Research Collaboratory for Structural Bioinformatics (RCSB) protein data bank (Berman et al., 2000). For 2UXA, chains A and C were used, and for the K759M/T765K mutant structure, chains A and B were used. Zinc ions were removed in both cases before simulation setup, and for all simulations based on the 2UXA structure, the R764G mutation was introduced. Missing atoms were added in PyMOL (The PyMOL Molecular Graphics System, Version 1.4, Schrödinger) and missing residues were added using Modeller Version 9.12 (Sali and Blundell, 1993). The LBD dimer was solvated in a cubic water box with dimensions (100 Å)^3 using the TIP3P water model (Jorgensen et al., 1983), and subsequently the system was neutralized and 150 mM NaCl or LiCl was added. Mutations were imposed manually prior to simulation setup, either by editing/deleting atoms in the pdb-file or by using the mutate function of PyMOL (The PyMOL Molecular Graphics System, Version 1.4, Schrödinger) and adjusting the side chain rotamer.

For MD simulations, the systems were first energy minimized until the maximum force on an atom was less than 100 kJ/mol/nm. Following energy minimization, a 200 ps restrained simulation with position restraints on protein heavy-atoms with a force constant of 1000 kJ mol^{-1} nm^{-2} was performed in the NVT ensemble with a temperature of 300 K maintained by a Berendsen thermostat (Berendsen et al., 1984). Periodic boundary conditions were employed and van der Waals interactions were cut off at 10 Å. Long-range electrostatics were accounted for by the Particle-Mesh Ewald method (Essmann et al., 1995). All bonds were treated as constraints using the LINCS algorithm (Hess, 2008), allowing a time step of 2 fs. Subsequently, another 200 ps restrained simulation was performed as above but in the NPT ensemble at a pressure of 1 bar, maintained by a Berendsen barostat (Berendsen et al., 1984). Following this, 100 ns of production run were performed.

X-ray crystallography.

During protein purification, cell pellets were incubated in high sucrose buffer (20 % (w/v) sucrose, 25 mM HEPES pH 8.0, 5 mM EDTA, 0.25 mg/ml lysozyme) for 45 min at room temperature, spun (2,000x g, 30 min, 4°C) and the pellets frozen at -80°C. These were thawed into 25 mM HEPES pH 7.5, 150 mM NaCl, 5mM L-Glu, 0.25 U/ml benzonase (Sigma), incubated (30 min at room temperature) and spun (18,500x g, 30 min, 4°C). Protein-containing supernatant was then purified (see main Experimental Procedures).

Crystals were grown in hanging-drops by mixing purified protein (5-10 mg/ml in 25mM HEPES, 150 mM NaCl, 5 mM L-Glu) in a 1:1 ratio with well solution containing either lithium (20-22 % PEG 4,000, 200 mM lithium sulfate, 100 mM acetate pH 5.0; grown at 6°C) or zinc ions (12-15 % PEG 8,000, 200 mM zinc acetate, 100 mM MES pH 6.0; grown at 23°C). Crystals grew in 1-3 weeks and were cryo-protected by briefly soaking in well solution containing 20-

22.5 % glycerol prior to plunge-freezing in liquid N_2. Diffraction limits were chosen based on a combination of $I / \sigma I > 1$, CC(1/2) > 0.5, and completeness in the outer shell > 90%. Molecular replacement in PHASER used the 2UXA GluA2 (flip) LBD structure as a model, modified with residues K759 and T765 truncated to Ala. Ramachandran statistics were 99.2/0.8/0.0 (% favored/allowed/outlier) for the zinc form and 99.0/1.0/0.0 for the lithium form.

SUPPLEMENTAL REFERENCES

Berman, H.M., Westbrook, J., Feng, Z., Gilliland, G., Bhat, T.N., Weissig, H., Shindyalov, I.N., and Bourne, P.E. (2000). The Protein Data Bank. Nucleic Acids Res 28, 235-242.

Bowie, D., Lange, G.D., and Mayer, M.L. (1998). Activity-dependent modulation of glutamate receptors by polyamines. J Neurosci 18, 8175-8185.

Essmann, U., Perera, L., Berkowitz, M.L., Darden, T., Lee, H., and Pedersen, L.G. (1995). A Smooth Particle Mesh Ewald Method. J Chem Phys 103, 8577-8593.

Geiser, M., Cebe, R., Drewello, D., and Schmitz, R. (2001). Integration of PCR fragments at any specific site within cloning vectors without the use of restriction enzymes and DNA ligase. BioTechniques 31, 88-90, 92.

Hess, B. (2008). P-LINCS: A parallel linear constraint solver for molecular simulation. J Chem Theory Comput 4, 116-122.

Jorgensen, W.L., Chandrasekhar, J., Madura, J.D., Impey, R.W., and Klein, M.L. (1983). Comparison of Simple Potential Functions for Simulating Liquid Water. J Chem Phys 79, 926-935.

Lomeli, H., Mosbacher, J., Melcher, T., Hoger, T., Geiger, J.R., Kuner, T., Monyer, H., Higuchi, M., Bach, A., and Seeburg, P.H. (1994). Control of kinetic properties of AMPA receptor channels by nuclear RNA editing. Science 266, 1709-1713.

Nayeem, N., Zhang, Y., Schweppe, D.K., Madden, D.R., and Green, T. (2009). A nondesensitizing kainate receptor point mutant. Mol Pharmacol 76, 534-542.

Rozov, A., Zilberter, Y., Wollmuth, L.P., and Burnashev, N. (1998). Facilitation of currents through rat Ca2+-permeable AMPA receptor channels by activity-dependent relief from polyamine block. J Physiol 511 (Pt 2), 361-377.

Sali, A., and Blundell, T.L. (1993). Comparative protein modelling by satisfaction of spatial restraints. J Mol Biol 234, 779-815.

Semenov, A., Moykkynen, T., Coleman, S.K., Korpi, E.R., and Keinanen, K. (2012). Autoinactivation of the stargazin-AMPA receptor complex: subunit-dependency and independence from physical dissociation. PLOS ONE 7, e49282.
