# Supplementary material for: Distinct Structural Pathways Coordinate the Activation of AMPA Receptor-Auxiliary Subunit Complexes
Source: Neuron. 2016 Mar 16;89(6):1264–76. doi: 10.1016/j.neuron.2016.01.038 (PMC4819453; doi:10.1016/j.neuron.2016.01.038)
Supplement: Document S2. Article plus Supplemental Information [file mmc6.pdf]

Neuron
Article

Distinct Structural Pathways Coordinate the Activation of AMPA Receptor-Auxiliary Subunit Complexes

Highlights

- Two distinct structural motifs control the time course of AMPA receptor gating
- Intraprotein electrostatic interactions govern gating by pore-forming subunits
- Auxiliary subunits act at a distinct site to prolong channel activity
- Intra- and interprotein interactions coordinate signaling by AMPA receptor complexes

Authors

G. Brent Dawe, Maria Musgaard, Mark R.P. Aurousseau, Naushaba Nayeem, Tim Green, Philip C. Biggin, Derek Bowie

Correspondence

tpgreen@liverpool.ac.uk (T.G.),
philip.biggin@bioch.ox.ac.uk (P.C.B.),
derek.bowie@mcgill.ca (D.B.)

In Brief

Combining electrophysiology, molecular dynamics simulations, and X-ray crystallography, Dawe et al. identify two distinct structural motifs that coordinate the gating of AMPA receptor-auxiliary subunit complexes, highlighting the importance of intra- and interprotein interactions in fast excitatory signaling.

Accession Numbers

5FTH
5FTI

Dawe et al., 2016, Neuron *89*, 1264–1276
March 16, 2016 ©2016 The Authors
http://dx.doi.org/10.1016/j.neuron.2016.01.038

Neuron
Article

Distinct Structural Pathways Coordinate the Activation of AMPA Receptor-Auxiliary Subunit Complexes

G. Brent Dawe,[1,2,5] Maria Musgaard,[3,5] Mark R.P. Aurousseau,[2,5] Naushaba Nayeem,[4] Tim Green,[4,6,*] Philip C. Biggin,[3,6,*] and Derek Bowie[2,6,*]
[1]Integrated Program in Neuroscience, McGill University, Montréal, QC H3A 2B4, Canada
[2]Department of Pharmacology and Therapeutics, McGill University, Montréal, QC H3G 1Y6, Canada
[3]Department of Biochemistry, University of Oxford, Oxford OX1 3QU, UK
[4]Department of Pharmacology, University of Liverpool, Liverpool L69 3BX, UK
[5]Co-first author
[6]Co-senior author
*Correspondence: tpgreen@liverpool.ac.uk (T.G.), philip.biggin@bioch.ox.ac.uk (P.C.B.), derek.bowie@mcgill.ca (D.B.)
http://dx.doi.org/10.1016/j.neuron.2016.01.038
This is an open access article under the CC BY license (http://creativecommons.org/licenses/by/4.0/).

SUMMARY

Neurotransmitter-gated ion channels adopt different gating modes to fine-tune signaling at central synapses. At glutamatergic synapses, high and low activity of AMPA receptors (AMPARs) is observed when pore-forming subunits coassemble with or without auxiliary subunits, respectively. Whether a common structural pathway accounts for these different gating modes is unclear. Here, we identify two structural motifs that determine the time course of AMPAR channel activation. A network of electrostatic interactions at the apex of the AMPAR ligand-binding domain (LBD) is essential for gating by pore-forming subunits, whereas a conserved motif on the lower, D2 lobe of the LBD prolongs channel activity when auxiliary subunits are present. Accordingly, channel activity is almost entirely abolished by elimination of the electrostatic network but restored via auxiliary protein interactions at the D2 lobe. In summary, we propose that activation of native AMPAR complexes is coordinated by distinct structural pathways, favored by the association/dissociation of auxiliary subunits.

INTRODUCTION

Voltage- and ligand-gated ion channels are signaling complexes that are often assembled from both regulatory and pore-forming subunits (Catterall et al., 2006; Jackson and Nicoll, 2011; Trimmer, 2015). AMPA-type (AMPAR) ionotropic glutamate receptors (iGluRs) are composed of pore-forming GluA1–GluA4 subunits (Dingledine et al., 1999) that coassemble with a variety of auxiliary proteins, including the transmembrane AMPAR receptor regulatory protein (TARP) and cornichon (CNIH) families (Jackson and Nicoll, 2011; Schwenk et al., 2009; Tomita et al., 2003), as well as CKAMP44 (von Engelhardt et al., 2010) and SynDIG1 (Kalashnikova et al., 2010), among others (Haering et al., 2014). Each pore-forming subunit possesses four principal domains, with the extracellular amino-terminal domain (ATD) controlling assembly and trafficking (Gan et al., 2015; Greger et al., 2007) and the ligand-binding domain (LBD) providing a bilobed agonist-binding pocket (Dawe et al., 2015). Meanwhile, the three transmembrane helices and re-entrant loop form the central pore domain, which governs cation selectivity and channel block (Huettner, 2015) and connects to the short, intracellular carboxyl-terminal domain (CTD). Once assembled, the native AMPAR is a homo- or heteromeric tetramer (Sobolevsky et al., 2009) with a variable stoichiometry of TARPs (Hastie et al., 2013) that may include additional CNIH subunits (Herring et al., 2013; Jackson and Nicoll, 2011). Understanding these interactions has been an area of intense study in recent years, especially as TARPs and CNIHs have been shown to directly affect the functional behavior of native AMPARs as well as synaptic plasticity mechanisms (Jackson and Nicoll, 2011). Exactly how pore-forming and auxiliary subunits work together to achieve this, however, remains to be established.

Since TARPs and CNIHs are transmembrane proteins, interactions with AMPARs are expected to rely upon their proximity in the plasma membrane. Interestingly, protein-protein interactions of this nature can be short- and long-lived. Autoinactivation of neuronal AMPARs is thought to reflect the rapid, millisecond-scale dissociation of AMPAR-TARP complexes mediated by receptor desensitization (Constals et al., 2015; Morimoto-Tomita et al., 2009). In contrast, single-channel analysis of AMPAR-TARP fusion proteins has revealed less frequent transitions between distinct gating modes of high and low open-channel probability (P_{open}) (Zhang et al., 2014) that are also thought to represent TARP-coupled and TARP-uncoupled forms of the receptor complex, respectively (Howe, 2015). The occurrence of distinct gating behavior raises the question as to how auxiliary subunits mediate their effects on AMPAR gating. One possibility is that agonist-binding triggers channel activation through a single set of structural interactions that is modulated when pore-forming subunits are associated with auxiliary subunits.

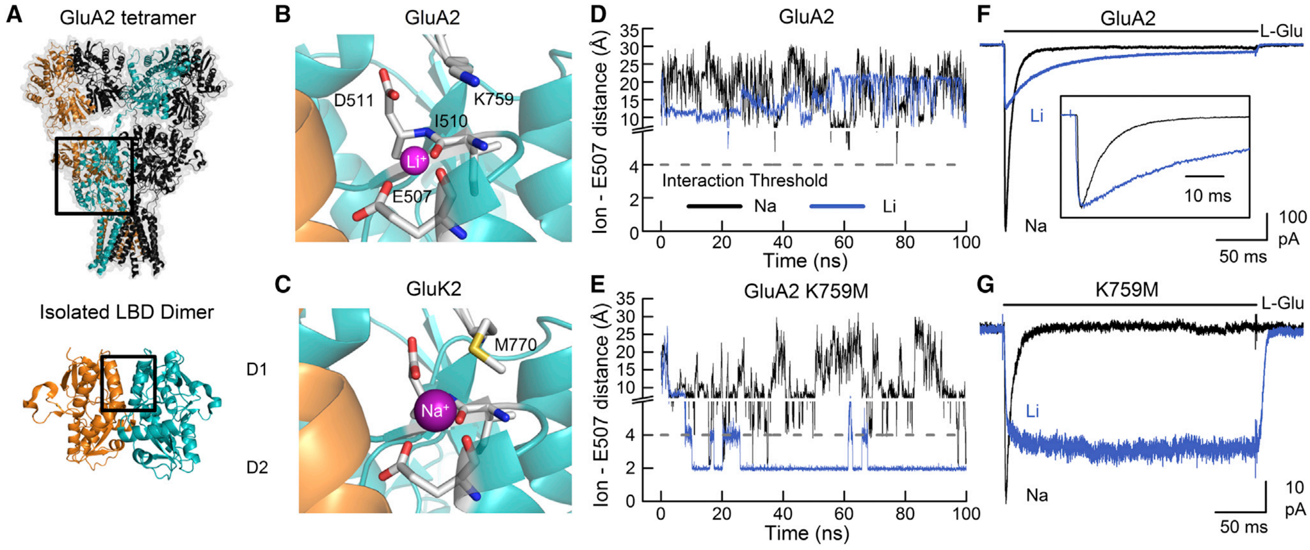

Figure 1. Lithium Modulates GluA2 Responses by Binding at the LBD Apex

(A) Crystal structure of the wild-type GluA2 tetramer (top, PDB: 3KG2; Sobolevsky et al., 2009) and isolated LBD dimer (bottom, PDB: 1FTJ; Armstrong and Gouaux, 2000).

(B and C) Illustration of the GluA2 (B) (PDB: 4IGT; Assaf et al., 2013) and GluK2 (C) (PDB: 2XXR; Nayeem et al., 2011) LBD dimer interfaces showing lithium and sodium ions, respectively, bound at a conserved electronegative pocket.

(D and E) Minimum distance between the nearest sodium or lithium ion and either sidechain oxygen atom found on residue Glu507 of chain A of wild-type GluA2 (D) or the K759M mutant (E). An interaction was deemed to occur when the cation was within 4 Å of an oxygen atom. In total, two 100 ns simulations were conducted in LiCl for each receptor, as well as three or four 100 ns simulations in NaCl for K759M and wild-type GluA2, respectively.

(F and G) Typical current responses elicited by 10 mM L-Glu on wild-type GluA2 (F) (patch number 140225p10) or K759M mutant (G) (patch number 140314p4) receptors in external solutions comprised of either NaCl or LiCl. Responses were also scaled to the same peak amplitude (inset).

Alternatively, auxiliary subunits may integrate other allosteric sites into the activation process, depending on how they are functionally coupled to AMPAR complexes.

Here, we have designed experiments to delineate between these two possibilities. Our data identify a network of intersubunit atomic bonds at the apex of the LBD that are critical to channel activation with pore-forming AMPAR subunits. This network can be stabilized by occupancy of an electronegative pocket that is conserved between AMPARs and kainate-type iGluRs (KARs). Disruption of the apical network abolishes almost all AMPAR gating, though coassembly with auxiliary subunits rescues function because of interactions relayed through the lower, D2 lobe of the LBD. Thus, while it is likely that a common mechanism ultimately triggers opening of the channel pore, we propose that channel activation of native AMPAR complexes is coordinated by pathways originating from distinct structural interactions. One interaction is LBD apex dependent and contained within pore-forming subunits, while the other is apex independent, stemming from the association of AMPARs and auxiliary subunits.

RESULTS

A Conserved Cation Pocket at the AMPAR and KAR LBD Dimer Interface

The topology of the iGluR tetramer is highly conserved between the AMPAR and KAR subfamilies, including the LBD, whose upper (D1) and lower (D2) lobes form the agonist-binding cleft (Figure 1A). AMPARs and KARs also possess an extensive network of electrostatic and hydrophobic interactions along the D1-D1 interface between subunits (Horning and Mayer, 2004) (Figures 1B and 1C), raising the question of their role in iGluR gating. In addition, KARs possess both sodium and chloride ion-binding pockets at the apex of this interface, which are critical for channel gating (Bowie, 2010). In GluK2 KARs, occupancy of the cation-binding pocket (Figure 1C) is required for activation (Wong et al., 2006), with the time course of channel activity regulated by the residence time of bound sodium (Dawe et al., 2013). Curiously, although AMPARs have been considered cation independent (Bowie, 2002), lithium has been modeled at this site in two X-ray crystal structures of the GluA2 LBD, including one determined at 1.24 Å resolution (Figure 1B) (Assaf et al., 2013) that exhibits many of the structural hallmarks of the KAR cation-binding pocket (Figure 1C). Because lithium is frequently present in crystallization buffers for the GluA2 LBD (Green and Nayeem, 2015), we sought to determine if the lithium site is artifactual, with little impact on AMPAR gating, or whether lithium binding under experimental conditions can modulate gating behavior.

To determine whether occupancy of the putative cation pocket affects AMPAR gating, molecular dynamics (MD) simulations were first performed to determine the residence time of lithium ions at wild-type GluA2 AMPARs (Figure 1D). Simulations were performed in either 150 mM NaCl or LiCl without initial occupancy of the pocket, enabling a prediction of whether cations readily bind to the site. When the distance between Glu507

(Figure 1B) and the closest sodium or lithium ion was monitored over a 100 ns simulation, little meaningful interaction occurred (Figure 1D). The average frequency of interactions below 4 Å, taken as the cutoff value for intermolecular electrostatic interactions, was 0.4% in NaCl and 5.2% in LiCl, when the two binding sites of the dimer were considered. One factor that might explain the low propensity for cation binding is the contribution of Lys759 (Figure 1B), which often makes an intrasubunit projection toward the pocket and may compete with lithium ions for contact with electronegative residues. We therefore repeated the MD simulations, incorporating a mutation that replaced the positively charged Lys with a Met residue, as found in GluK2 KARs. As anticipated, lithium resided in the putative cation pocket for much longer periods of time (Figure 1E), confirming that removal of Lys759 impacts the ability of lithium to bind. Contact frequency between lithium and Glu507 averaged 52.1% of simulation time, while sodium binding remained infrequent at 1.9% (Movies S1 and S2, available online). Together, these data make the prediction that lithium binding to the apex of the GluA2 LBD would have measurable consequences on AMPAR gating, which would be more pronounced for GluA2 K759M receptors.

Accordingly, we performed cation substitution experiments during patch-clamp recordings to determine whether lithium modulates the gating behavior of wild-type and mutant GluA2 AMPARs. Membrane currents elicited by L-Glu in 150 mM NaCl at wild-type GluA2 and K759M receptors decayed rapidly with time constants of 6.9 ± 0.2 ms (n = 7; Figure 1F) and 9.9 ± 0.6 ms (n = 8; Figure 1G), consistent with MD simulations showing that sodium ions interact little with the electronegative residues of the cation pocket. The substitution of external NaCl with LiCl caused a dramatic slowing in the onset of desensitization (τ = 50.0 ± 3.4 ms; n = 7; $p < 0.0001$) for wild-type GluA2 (Figure 1F) and yielded a nondecaying phenotype (n = 6) in GluA2 K759M receptors (Figure 1G). In contrast, substitution with the larger monovalent cation potassium had minimal effect on decay kinetics of both wild-type and mutant GluA2 receptors (Figure S1). This suggests that access to the electronegative, "cation" pocket of AMPARs is restricted to ions of smaller ionic radius. Moreover, single-channel recordings revealed that external lithium prolongs the occurrence of channel openings prior to desensitization (Figure S1). Because the duration of this activity is affected by microscopic rates of channel opening and closing, as well as agonist unbinding and/or desensitization, we refer to channel activation/activity as the sum of these processes.

Taken together, our observations corroborate the idea that in 150 mM LiCl external solution, lithium ions can bind to an electronegative pocket in wild-type and mutant GluA2 AMPARs, sustaining channel activity in an analogous manner to sodium binding at KARs (Dawe et al., 2013). However, unlike sodium, the presence of lithium in the nervous system is typically negligible, and even during lithium treatment for bipolar disorder, effective serum concentrations range from 0.4 to 1.2 mM (Severus et al., 2008). When we supplemented our standard external recording solution with 1.5 mM LiCl, there was no significant change in GluA2 decay kinetics ($p = 0.82$; n = 5; data not shown), meaning we could not ascribe a physiological role to cation binding at the GluA2 LBD. Instead, we used lithium as an experimental tool to interrogate the structural interactions modulated by its binding and how these interactions shape the overall functional output of AMPARs.

GluA2 Activation Does Not Require Electronegative Pocket Occupancy

One question not addressed by the cation substitution experiments is whether wild-type GluA2 or K759M AMPARs gate in the absence of external ions, as described previously for GluK2 KARs (Dawe et al., 2013; Wong et al., 2006). The issue is especially relevant for K759M receptors, where our data already establish that removal of the positively charged Lys provides a favorable binding site for external lithium ions (Figures 1E and 1G). The idea that AMPARs with the K-M mutation may be rendered cation sensitive has been considered previously for GluA1 receptors, but it was not pursued further due to poor expression of the mutant (Wong et al., 2006). Using TIRF microscopy of GFP-tagged AMPARs, we confirmed that the equivalent K759M mutation in GluA2 did not prevent receptor expression at the plasma membrane (Figure S2). We therefore repeated experiments in external ion-free conditions for wild-type and mutant GluA2 receptors to determine their agonist responsiveness (Figure 2). In agreement with observations on GluA1 receptors, GluA2 AMPARs continued to be activated by L-Glu, even in the absence of external NaCl, establishing that GluA2 AMPAR gating is not dependent on external cations, unlike GluK2 KARs (Figures 2A and 2B). GluA2 K759M also continued to elicit membrane currents when external NaCl was removed (Figure 2C), and in this condition, both AMPARs produced outwardly rectifying current-voltage (I-V) plots that contrasted with the loss of the GluK2 response (Figures 2D–2F). These data demonstrate that while KARs require external cations to activate, GluA2 AMPARs require neither interactions with Lys759 in the wild-type receptor nor occupancy by cations in the K759M mutant. As such, additional interactions modulated by lithium binding at the electronegative pocket must be able to profoundly affect GluA2 AMPAR activation.

The Electronegative Pocket Acts through Intersubunit Contacts

Since the lithium binding site is quite distant from the channel pore, it remained unclear how lithium might influence LBD structure to stabilize the activated state of the receptor. To address this, we used MD simulations, which revealed that cation binding promotes rearrangements in the GluA2 K759M LBD dimer interface. Specifically, increasing the number of bound lithium ions shifted the distribution of predicted distances across the interface in a negative direction (Figures 3A and 3B). Because these distances were measured between two points at the apex of each D1 lobe, they are referred to as D1-D1 interface distances (Figure 3B). Nevertheless, lithium binding sites are fully contained within single subunits on each side of the interface, making it unlikely that lithium acts directly as an adhesive force between subunits. However, the ion is coordinated by Glu507, which forms electrostatic interactions across the interface with both Lys514 and Asn768 (Figure 3A). This prompted us to explore whether lithium modulates GluA2 current decay kinetics

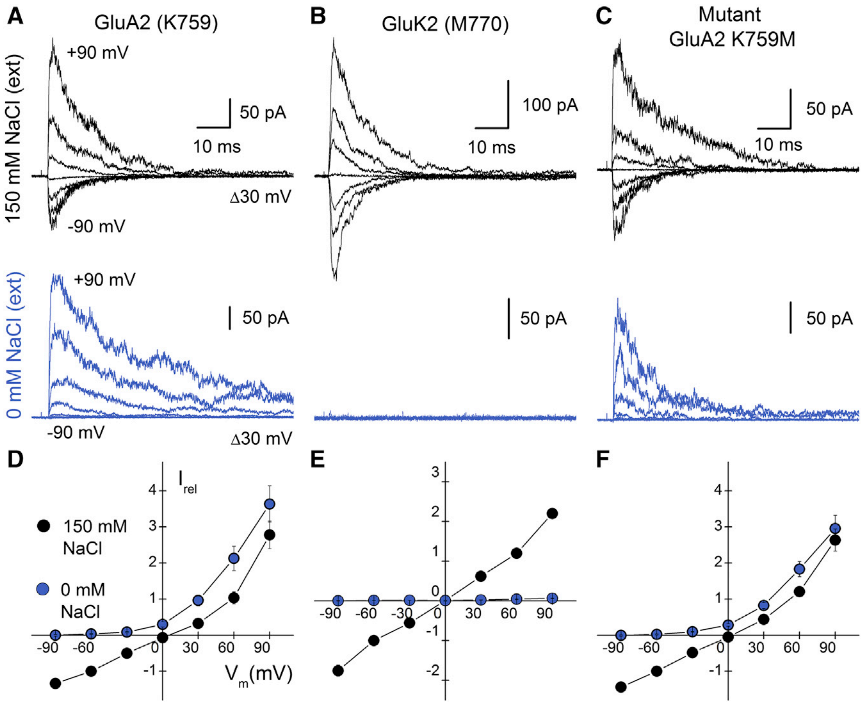

Figure 2. GluA2 K759M Exhibits Robust Activation in the Absence of External NaCl

(A–C) Membrane currents evoked by 1 (for KARs) or 10 mM (for AMPARs) L-Glu acting on wild-type GluA2 (A) and GluK2 (B), as well as GluA2 K759M mutant (C) receptors, in either 150 mM NaCl (top) or NaCl-free, sucrose-based (bottom) external solution (V_m = −90 to +90 mV, at 30 mV increments). For each receptor, the same patch was recorded in both ionic conditions. For wild-type GluA2 (patch number 140417p4) and the K759M mutant (patch number 140502p1), outward currents persisted at positive holding potentials, whereas GluK2 responses (patch number 140904p3) were abolished.

(D–F) Current-voltage plots in 0 mM (blue) and 150 mM (black) NaCl for wild-type GluA2 (D), GluK2 (E), and GluA2 K759M (F) receptors. Currents were normalized to responses at −60 mV in 150 mM NaCl. Data are mean ± SEM, from four (GluA2), three (GluK2), or six (K759M) independent experiments for each receptor.

by stabilizing intersubunit electrostatic interactions. We therefore removed these interactions in a K514M/N768T double mutant, where the mutated residues retain approximately the same bulkiness but lose their charge or ability to form the same crossdimer hydrogen bonds. This mutant exhibited currents that decayed with time constants of 8.4 ± 1.2 ms (n = 5) in NaCl and 6.9 ± 1.1 ms (n = 5) in LiCl (Figures 3C and 3D). The observation that decay kinetics were not significantly different between cation species (p = 0.26) stands in marked contrast to wild-type GluA2 (Figure 3D) and confirms that lithium modulation was abolished. Since it is possible that lithium binding was disrupted in GluA2 K514M/N768T, we used MD simulations to evaluate this possibility. MD data revealed no gross conformational changes to the LBD dimer and, moreover, reported that lithium ions interact with the pocket with a frequency similar to or greater than with wild-type GluA2 (Figure S3). Taken together, our data indicate that experimental concentrations of external LiCl (i.e., 150 mM) influence intersubunit electrostatic contacts at the apex of the LBD dimer interface, thereby stabilizing the activated conformation of the receptor. To explore this idea further, we investigated whether strengthening the apex of the LBD dimer interface could sustain AMPAR activation.

Engineering an Intersubunit Tether to Sustain Channel Activation

In order to incorporate an additional electrostatic interaction across the D1-D1 interface, we used a Thr765 to Lys mutation to introduce a charged tether onto residues forming the opposing electronegative pocket (for additional rationale, see Figure S4). Alone, this mutation had little functional effect, but coupled with the K759M mutation (K759M/T765K), current decay slowed several fold, and the additional mutation N768T (creating K759M/T765K/N768T, or MKT) yielded nondecaying current responses (Figure 4A). Consistent with this, single-channel events of GluA2 MKT were sustained throughout the 250 ms period of agonist application, in contrast to wild-type channels (Figures 4B and 4C). In both cases, current records were fit with four conductance levels of approximately 6, 12, 24, and 40 pS, with the P_{open} of GluA2 MKT estimated to be 0.62 ± 0.14 (n = 4) (Figure 4D). The occurrence of MKT channel closures in these conditions could be explained by the failure of the mutant Lys residue to form a sustained, crossdimer tether, enabling the LBD dimer to rupture.

In order to verify that a Lys tether had been introduced across the GluA2 LBD dimer, we attempted structural analysis of the MKT mutant. However, protein expression levels were too low to obtain diffracting crystals. In contrast, crystals of the GluA2 K759M/T765K LBD were successfully grown in the presence of zinc, and a dataset was collected from a single crystal at 2.9 Å resolution (Table S1). Three protomers were present in the asymmetric unit, of which chains A and B formed a canonical dimer, and the third, C, formed a dimer with its symmetry-related counterpart. In each dimer (A:B and C:C′) electron density was visible for both the mutant Met and Lys residues, and the latter residue was spanning the dimer interface as predicted (Figures 4E and S5). Electrostatic interactions were formed between the amine group on residue 765 (i.e., T765K) and the sidechain carboxyl group of Asp511, as well as the backbone oxygen atom of Ile510 (Figure 4E). In addition to these contacts, there was a general shift in the dimer conformation, with the apical residues having moved closer together relative to structures of wild-type GluA2, forming a more extensive, contiguous interface (Figure 4F).

Consistent with functional recordings of GluA2 K759M/T765K (Figure 4A), our structural data also suggest that the crossdimer tether does not persist indefinitely. First, an additional crystal structure grown in the presence of lithium (Table S1) revealed

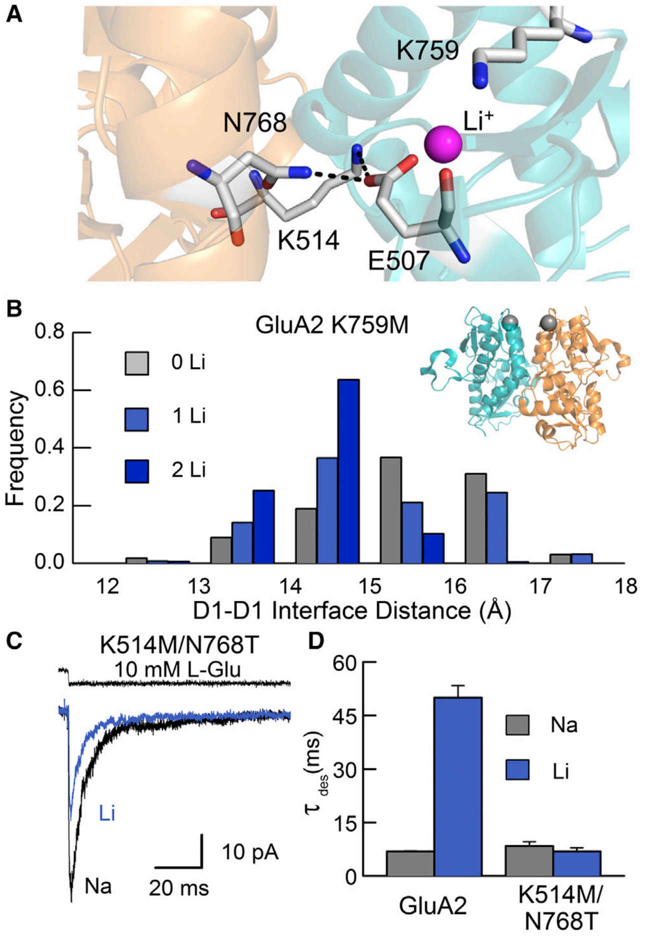

Figure 3. Lithium Modulation Is Mediated by Crossdimer Electrostatic Contacts

(A) Image of an intersubunit salt bridge and hydrogen bond adjacent to the lithium binding site (PDB: 4IGT; Assaf et al., 2013). Residues Lys514 and Asn768 are from chain A, while Glu507 and Lys759 are from chain B.

(B) Intersubunit distance across the apex of the GluA2 LBD, relative to the number of lithium ions occupying the two cation pockets, measured during 100 ns MD simulations (two repeats) of GluA2 K759M in LiCl. Distances were measured between the gray spheres (inset, right), which represent a center of mass for Cα atoms of residues 508–510 and 759–765.

(C) Typical current responses to L-Glu obtained from the GluA2 K514M/N768T mutant (patch number 140718p4), recorded in external NaCl and LiCl. The top trace (black) shows the junction current, recorded with an open patch pipette after the experiment to monitor the profile of solution exchange.

(D) Plot of current decay time constants (τ_{des}) for wild-type GluA2 and K514M/N768T receptors. Data are mean ± SEM, from seven (wild-type GluA2) or five (K514M/N768T) independent patch experiments for each receptor.

that the electronegative pocket was partially occupied by a lithium ion (Figures S4 and S5) and not the opposing Lys residue. Second, in MD simulations of both the double- and triple-mutant receptors, the T765K residue failed to make continuous contact with the electronegative pocket (Figures 4G and 4H; Movies S3 and S4). Overall, these structural and functional data support the premise that the Lys tether is not a permanent feature of the T765K mutant series. However, the MKT mutation makes tethering more favorable, likely because the replacement of Asn by the smaller Thr at position 768 reduces steric block, thereby allowing subunits within each LBD dimer to come closer together. As explained below, we explored the opposite effect of dimer crosslinking by determining if elimination of electrostatic interactions at the apex of the LBD dimer interface would disrupt GluA2 AMPAR functionality.

Removal of an Electrostatic Network Disrupts Gating by Pore-Forming Subunits

Although the addition of new crossdimer interactions (e.g., GluA2 MKT) can sustain GluA2 gating, the mutation of other interface residues has been shown to curtail channel activity. For example, the individual conversion of residues Glu507, Lys514, and Asn768 at the apex of the dimer interface (Figure 5A) to Ala speeds desensitization (Horning and Mayer, 2004). Of these residues, Glu507 and Lys514 form a salt bridge (Figure 5A). Interestingly, the two residues are conserved in AMPARs and KARs, but not NMDARs (Figure S6), suggesting that different sets of interactions regulate their slow time course of activation. However, because both Asn768 and Phe512 (via a backbone oxygen atom) can also contribute to the electrostatic network in GluA2, we evaluated the effect of completely disrupting this network using the triple-mutant GluA2 E507A/K514A/N768A (i.e., GluA2 AAA). On this note, mean peak current responses elicited by GluA2 AAA (94.5 ± 28.5 pA; n = 7) were depressed by almost 10-fold compared to wild-type GluA2 receptors (928 pA ± 317 pA; n = 12) (Figures 5B and 5C). In addition, the onset of desensitization was almost 10-fold faster for GluA2 AAA (τ = 0.74 ± 0.06 ms; n = 7) versus wild-type GluA2 (τ = 6.1 ± 0.2 ms; n = 7) (Figure 5D). The diminished functionality of the GluA2 AAA mutant demonstrates that the network of electrostatic interactions at the apex of the LBD dimer interface is a key structural element mediating channel gating by pore-forming AMPAR subunits.

Appreciating that the positive allosteric modulator cyclothiazide (CTZ) binds to the bottom of the D1-D1 interface (Sun et al., 2002), we tested whether AMPAR functionality could be recovered when CTZ was present. CTZ restored the responsiveness of the GluA2 AAA mutant, causing an 8.5 ± 1.0-fold (n = 7) increase in the peak response. In marked contrast, CTZ potentiated wild-type GluA2 currents to a significantly lesser extent of 1.3 ± 0.03-fold (n = 11; $p < 0.001$; Figures 5B, 5C, and 5E). However, since functionality can be restored by CTZ, we conclude that, under certain circumstances, other interactions are capable of coordinating channel gating independent of the LBD apex region. To explore this further, we tested whether the functionality of GluA2 AAA could be rescued by coexpression with auxiliary subunits.

Auxiliary Subunits Rescue Functionality of the GluA2 AAA Mutant

To test the effect of TARP or CNIH protein association on GluA2 AAA, we coexpressed the mutant receptor with either γ2 or γ7 TARP subunits or CNIH-3 (Figure 6). To control for the effect of TARPs and/or CNIHs on AMPAR trafficking (Jackson and Nicoll, 2011), we used the potentiation of peak L-Glu responses by CTZ as an estimate of P_{open} (Cho et al., 2007), or gating ability, in each

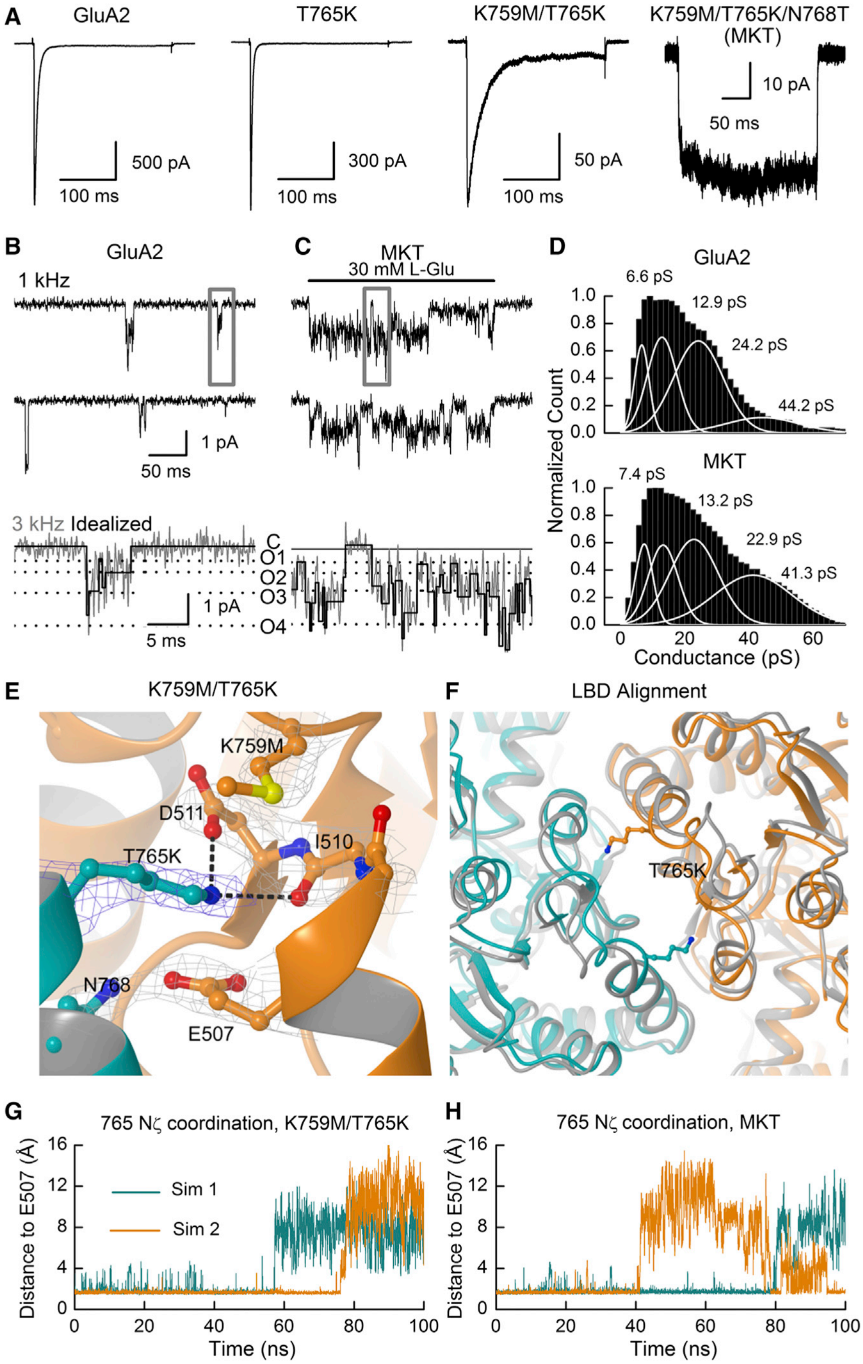

Figure 4. Structural and Functional Data Show T765K Can Act as a Crossdimer Tether

(A) Typical current responses to 10 mM L-Glu for a series of GluA2 mutants engineered to form a crossdimer tether. Wild-type GluA2 (patch number 130221p5) and mutants T765K (patch number 130617p4), K759M/T765K (patch number 130618p6), and K759M/T765K/N768T, or MKT (patch number 130917p6), are shown left to right.

(B and C) Unitary channel activity evoked by 30 mM L-Glu for wild-type GluA2 receptors in equilibrium conditions (B) (patch number 131212p7) and the triple-mutant MKT (C) (patch number 140124p1) during a 250 ms agonist application. Typical records are shown low-pass filtered at 1 kHz (top) or the 3 kHz threshold used to fit channel openings (bottom), expanded from gray box above. Horizontal dotted lines correspond to the conductance levels of open states (O1–O4) fit in (D).

(D) Distributions of conductance levels from idealized records of wild-type GluA2 (top) or GluA2 MKT (bottom) fit with four Gaussian functions (white lines). Openings were analyzed using four patch recordings for each receptor.

(E) View of protomers A (orange) and B (teal) from the K759M/T765K structure, zinc form, showing T765K tethering onto electronegative residues on the opposing subunit. Electron density ($|2F_{obs} - F_{calc}|\alpha_{calc}$, contoured at 1.3σ) is shown around the displayed side chains only. Interactions between the sidechain amine group of residue 765 and atoms in protomer A are shown as dashed lines.

(F) Top view of an alignment between wild-type GluA2 (gray; PDB: 1FTJ; Armstrong and Gouaux, 2000) and K759M/T765K (orange/teal) LBD dimers.

(G and H) Minimum distance between the amine-group nitrogen atom on the mutant Lys (introduced on chain B) and either sidechain oxygen atom found on residue Glu 507 (on chain A) for K759M/T765K (G) and the MKT mutant (H). Simulations were performed using the GluA2 K759M/T765K LBD dimer, while the N768T mutation was introduced atop this structure to simulate GluA2 MKT. Two repeats are shown for each mutant.

condition. Large membrane currents were elicited from GluA2 AAA receptors when coexpressed with either TARP or CNIH subunits, contrasting with the AAA mutant expressed alone (Figures 6A–6D). Moreover, peak current potentiation of GluA2 AAA responses by CTZ was significantly reduced to 1.5- to 3-fold when receptors were coexpressed with γ2, γ7, or CNIH-3 subunits ($p < 0.002$ in all cases), though still higher than observed with wild-type receptors (Figure 6E). This finding reaffirms our hypothesis that auxiliary subunits are capable of coordinating channel gating of pore-forming subunits, independent of the network of electrostatic interactions at the LBD apex region. Also, desensitization kinetics of GluA2 AAA were markedly faster than wild-type receptors when coexpressed with TARPs γ2 and γ7 (Figures 6F and 6G). Auxiliary subunits therefore do not fully rescue the gating deficits of GluA2 AAA and most likely coordinate channel gating in synchrony with the apex region of the AMPAR LBD dimer interface. As a consequence, AMPAR channel gating is coordinated by apex-dependent and apex-independent interactions. The former are comprised of an intraprotein electrostatic network that mediates the activation of pore-forming subunits, while the latter depends upon interactions that become available upon the association of auxiliary subunits.

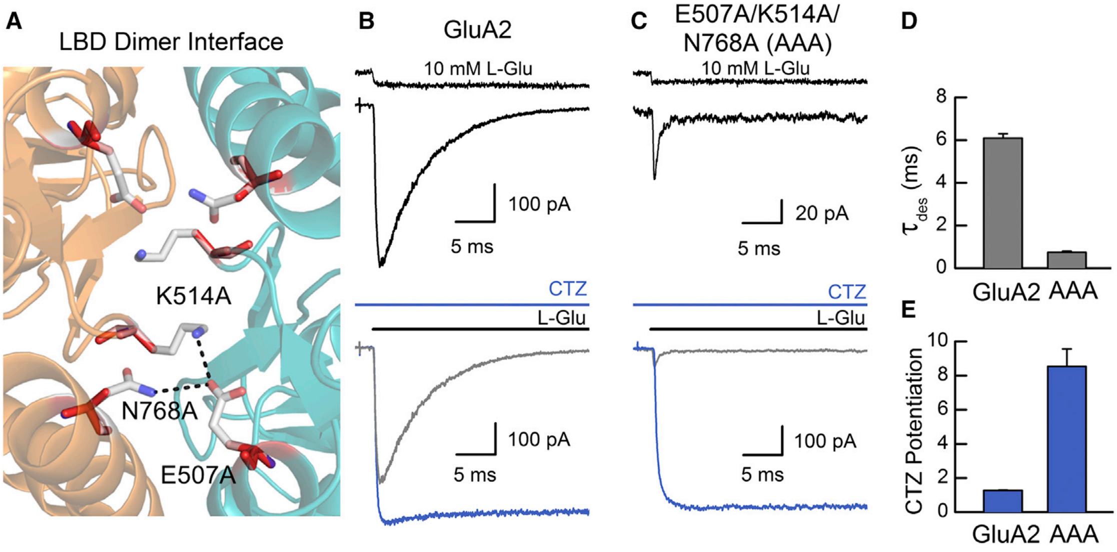

Figure 5. Truncation of Key Residues at the LBD Apex Produces Poorly Functioning Receptors

(A) Top view of the GluA2 LBD dimer interface (PDB: 1FTJ; Armstrong and Gouaux, 2000), showing charged and polar residues (faint gray) that were mutated to Ala (red). Labeled residues Lys514 and Asn768 are from chain A, while Glu507 is from chain B.

(B and C) Typical current responses of wild-type GluA2 (B) (patch number 130305p7) and the E507A/K514A/N768A, or AAA, mutant (C) (patch number 151005p6) to L-Glu before (top, black; bottom, gray) and during (bottom, blue) exposure to cyclothiazide (CTZ), which attenuates desensitization. The uppermost trace (black) shows the junction current, recorded with an open patch pipette after the experiment to monitor the profile of solution exchange.

(D) Average time constants of current decay (τ_{des}) for wild-type GluA2 and the AAA mutant. Data are mean ± SEM, from seven (wild-type GluA2 and GluA2 AAA) independent patch experiments.

(E) CTZ potentiation of wild-type GluA2 and AAA mutant peak currents. Data are mean ± SEM, from eleven (wild-type GluA2) or seven (GluA2 AAA) independent patch experiments.

TARPs Modulate the Duration of AMPAR Gating by Interactions on the D2 Lobe

In order to pinpoint the site(s) where auxiliary proteins modulate AMPAR gating, we first compared the sequence of AMPAR and KAR LBDs. Since KARs do not bind TARPs (Chen et al., 2003), we reasoned that a sequence alignment would identify residues unique to AMPARs that may form functional interactions with auxiliary subunits. The most promising site was a Lys-Gly-Lys, or KGK motif (residues 718–720), situated on the lower, D2 lobe of the GluA2 LBD, which is conserved among all AMPAR subunits (Figures 7A and 7B). The KGK motif faces outward, where an auxiliary subunit might be expected to reside, based on previous cryo-EM (electron microscopy) images of native AMPARs (Nakagawa et al., 2005). These three amino acids were therefore substituted with the single Asp residue (termed "3D" mutation) found in GluK1-3 KARs, where two residues are lost (Figure 7B). Importantly, the GluA2 3D mutant receptor had similar kinetic properties to wild-type GluA2, with deactivation and desensitization time constants of 0.53 ± 0.05 ms (n = 5) and 6.2 ± 0.5 ms (n = 5), respectively, demonstrating that this site has a minimal effect on channel gating mediated solely by pore-forming subunits.

To study the functional impact of the 3D mutant on TARP-dependent gating, we used a GluA2/γ2 fusion protein to constrain subunit stoichiometry and also to prevent any confounding effect of disrupting AMPAR-TARP association. We then evaluated the 3D mutant by investigating three sets of AMPAR properties known to be regulated by TARP association: the time course of channel activation (Priel et al., 2005), apparent agonist efficacy (Turetsky et al., 2005), and the degree of polyamine channel block (Soto et al., 2007). First, we examined the time course of L-Glu-induced channel activation by measuring both deactivation and desensitization kinetics (Figures 7C and 7D). We also assessed the degree of equilibrium desensitization by measuring the equilibrium/peak response ratio (Figure 7E). Second, we examined apparent agonist efficacy by using CTZ potentiation as an indicator of peak P_{open} (Cho et al., 2007) and measuring the KA/L-Glu current ratio (Figure S7). Finally, we analyzed the affinity and voltage dependency of polyamine channel block, which was determined using 100 μM internal spermine (Figure S7).

When incorporated into the wild-type GluA2/γ2 fusion receptor, the 3D mutation accelerated deactivation and desensitization kinetics from 3.2 ± 0.4 ms (n = 9) and 45.7 ± 6.8 ms (n = 11), respectively, to 1.1 ± 0.1 ms (n = 8) and 12.7 ± 1.2 ms (n = 8), respectively (Figures 7C and 7D). Notably, the deactivation (τ = 0.67 ± 0.07 ms; n = 7) and desensitization (τ = 9.5 ± 0.4 ms; n = 7) time constants of GluA2 3D coexpressed with γ2 were statistically indistinguishable from GluA2 expressed alone (p = 0.95 and p = 0.29, respectively; Figures 7F and 7G), suggesting that the 3D mutant almost completely abolishes the effects of γ2 on the time course of GluA2 channel activity. Likewise, the equilibrium/peak response (%) was also reduced from 16.7% ± 2.9% (n = 11) with GluA2/γ2 to 5.1% ± 1.2% (n = 8) with GluA2 3D/γ2 (Figure 7E), which was much closer to the equilibrium/peak response of GluA2 alone (Figures 7E and 7H). The reverse mutation in GluK2 KARs (i.e., Asp732 to Lys-Gly-Lys)

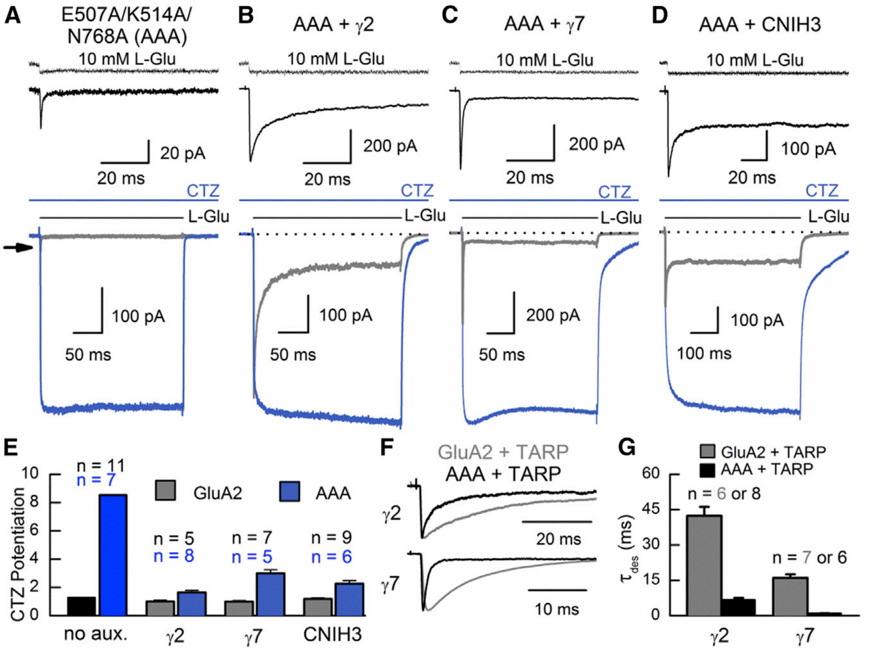

Figure 6. Coexpression of Auxiliary Subunits Rescues Function of the GluA2 AAA Mutant

(A–D) Behavior of GluA2 E507A/K514A/N768A, or AAA, receptors when expressed alone (A) (patch number 151008p10) or coexpressed with the TARP subunits γ2 (B) (patch number 140731p3) or γ7 (C) (patch number 141006p8), as well as the CNIH subunit CNIH-3 (D) (patch number 140926p5). Traces correspond to L-Glu-evoked responses prior to CTZ application (top, black; bottom, gray) or responses during (blue) CTZ exposure. The uppermost trace (black) shows the junction current, recorded with an open patch pipette after the experiment to monitor the profile of solution exchange. Arrow indicates the peak response of GluA2 AAA.

(E) CTZ potentiation of wild-type GluA2 and AAA mutant currents, tabulated in the presence or absence (no aux.) of different auxiliary subunits. Data are mean ± SEM, from the number of independent patch experiments indicated. Values with auxiliary subunits absent are as reported in Figure 5.

(F) Scaled comparison of wild-type GluA2 (gray) and AAA mutant (black) responses when coexpressed with TARP subunits γ2 (wild-type patch number 141006p3, AAA patch number 140721p3) and γ7 (wild-type patch number 141013p4, AAA patch number 141006p8).

(G) Time constants of current decay (τ_{des}) for wild-type GluA2 and GluA2 AAA coexpressed with TARP subunits γ2 or γ7. Data are mean ± SEM, from the number of independent patch experiments indicated.

produced no significant change in channel kinetics between the mutant receptor expressed alone or as a GluK2/γ2 fusion protein (data not shown), suggesting that these residues in the D2 lobe are not sufficient to confer functional TARP modulation of KARs. Taken together, our data identify the KGK motif as the critical structural element by which TARP γ2 prolongs the time course of AMPAR channel activation.

Interestingly, other functional properties of AMPARs modulated by TARPs, such as CTZ potentiation, KA/L-Glu current ratio, and polyamine channel block, were unchanged in the GluA2 3D/γ2 mutant receptor (for details, see Figure S7). These findings demonstrate that TARPs are still able to associate with the 3D mutant GluA2 subunits, despite the reduced modulation of channel decay kinetics. Importantly, these findings also show that the 3D site only accounts for a subset of all properties by which TARPs regulate AMPARs.

LBD Dimer Apex and the D2 Lobes Coordinate Channel Activation Independently

Because the 3D site profoundly attenuates the prolongation of channel activation by TARPs, we examined whether functional coupling between the D2 lobe and the TARP γ2 could account for the rescue of GluA2 AAA receptors by auxiliary subunits (Figure 6). To do this, the time course of channel activation of the double-site mutant, GluA2 AAA/3D, was compared in the presence and absence of TARP γ2 (Figure 8). In the absence of TARP subunits, there was no significant difference between desensitization time constants for GluA2 AAA and GluA2 AAA/3D (τ = 0.68 ± 0.10 ms; n = 6; p = 0.56; Figures 8A and 8B). Consistent with the phenotype of GluA2 AAA, the mean peak response of GluA2 AAA/3D was also small in amplitude (29.8 ± 8.6 pA; n = 7) and greatly potentiated by CTZ (17.0 ± 2.2-fold; n = 7; Figure 8B). However, when coexpressed with the γ2 subunit, the time constant of desensitization was about 3-fold faster (τ = 2.4 ± 0.3 ms; n = 7) for GluA2 AAA/3D than GluA2 AAA (τ = 6.6 ± 0.9 ms; n = 8; p = 0.002; Figures 8C–8E). The attenuation in γ2 modulation of the AAA mutant demonstrates that the 3D site is largely responsible for rescuing the time course of channel activation. Figure 8E summarizes how the coexpression of γ2 affects desensitization rates of the AAA and/or 3D mutant GluA2 receptors. Whether LBD apex interactions are present (i.e., wild-type GluA2) or absent (i.e., GluA2 AAA), the 3D mutation reduces TARP modulation of desensitization kinetics approximately 3-fold (Figure 8E). This suggests an independence of the LBD apex and D2 lobe in regulating the gating behavior of TARP-associated AMPARs. In summary, our data support a model where different sets of structural interactions determine the time course of activation of AMPAR-auxiliary subunit complexes (Figure 8F).

DISCUSSION

This study advances our understanding of AMPARs in two fundamental ways. First, we demonstrate that an evolutionarily conserved electrostatic network within the LBD apex is critical for the activation of pore-forming AMPAR subunits, which use it to generate rapid, millisecond-scale gating at central synapses. This network can be stabilized by the occupancy of an adjacent cation pocket, sustaining channel activation by a similar mechanism to sodium binding at KARs (Dawe et al., 2013).

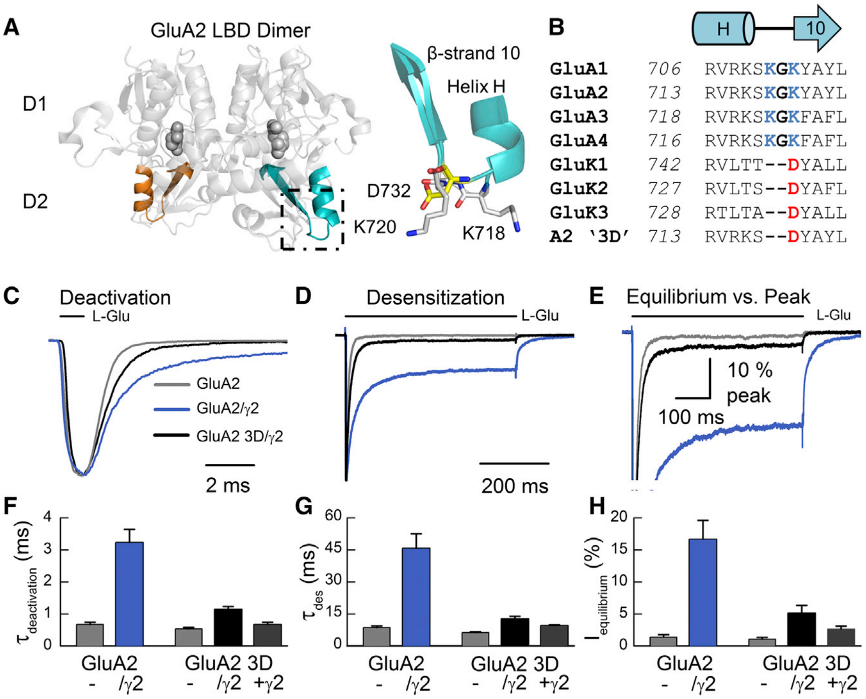

Figure 7. A Single D2 Mutation Attenuates TARP γ2 Modulation of GluA2 Current Decay

(A) View of the GluA2 LBD dimer (PDB: 1FTJ; Armstrong and Gouaux, 2000), highlighting the site of the 718–720 KGK to D (3D) mutation (in color, at left), between helix H and β strand 10 on the D2 lobe (at right). Mutated residues appear as in GluA2 (gray stick) or GluK2 (yellow stick) structures (PDB: 1FTJ or 2XXR; Nayeem et al., 2011).

(B) Sequence alignment of the 3D mutation site for rat AMPAR and KAR subunits.

(C and D) Scaled current responses of wild-type GluA2 (patch number 150317p2, gray), as well as GluA2/γ2 (patch number 150316p3, blue) and GluA2 3D/γ2 (patch number 150511p6, black) AMPAR-TARP fusion proteins to 1 ms (C) and 500 ms (D) applications of 10 mM L-Glu.

(E) Scaled equilibrium responses of wild-type GluA2 (patch number 150317p3, gray), as well as GluA2/γ2 (patch number 150316p3, blue) and GluA2 3D/γ2 (patch number 150511p6, black) AMPAR-TARP fusion proteins during a 500 ms L-Glu application.

(F–H) Mean time constants of current decay after a 1 ms L-Glu application ($\tau_{deactivation}$) (F) or in the continued presence of L-Glu (τ_{des}) (G), as well as mean equilibrium current amplitude, as a percentage of the peak response (H). Data are mean ± SEM, from the number of independent patch experiments that follows: eight (F) or nine (G and H) for GluA2, nine (F) or eleven (G and H) for GluA2/γ2, five (F–H) for GluA2 3D, eight (F–H) for GluA2 3D/γ2, and seven (F–H) for coexpressed GluA2 3D + γ2.

Although physiological cation species do not appear to regulate the GluA2 LBD apex, the near loss of channel activity after elimination of the electrostatic network indicates this region is one of the most important structural determinants of AMPAR gating. Accordingly, our observations reveal that for both KAR and AMPAR families, changes in only a few critical atomic interactions can drastically alter the time course of channel activation. Second, we show that pore-forming AMPAR subunits use different gating pathways when associated with and without auxiliary proteins. Although TARPs have been the focus of numerous studies in recent years, the structural interactions underpinning their modulation of AMPARs have remained largely unknown. Our data identify an important site at the D2 lobe of the GluA2 LBD, which mediates TARP prolongation of channel gating independently of interactions at the LBD apex. Because this motif does not affect other properties modulated by TARPs (i.e., agonist efficacy and permeation), we conclude that several discrete sites must act together to bring about the ensemble behavior of TARP-bound AMPARs.

An Evolutionarily Conserved Hotspot Governing KAR and AMPAR Activation

A key difference between KARs and other iGluRs subfamilies is that external cations are required for KAR activation, in addition to modulating their gating behavior (Bowie, 2002; Wong et al., 2006). Although AMPAR and KAR protein architecture is very similar, the ability of cations to modulate AMPARs has not been thoroughly studied. In part, this was due to the discrepancy between the KAR cation-binding pocket, which can bind monovalent cations of various sizes (Bowie, 2002; Plested et al., 2008), and the equivalent AMPAR site, where lithium binding was only recently observed (Assaf et al., 2013). Moreover, the gating kinetics of GluA1 AMPAR subunits lack modulation by cations (Bowie, 2002) and perhaps cannot bind lithium. It should be noted that a potentiation of GluA2 and GluA3 equilibrium currents by external lithium was reported in oocytes (Karkanias and Papke, 1999), and later experiments characterized an increase in native AMPAR P_{open} under similar conditions (Gebhardt and Cull-Candy, 2010). These observations are consistent with the behavior we observed in outside-out patch recordings; however, no structural mechanism was then ascribed to them.

By combining recordings of full-length GluA2 receptors with simulations of the LBD dimer, we were able to show that high experimental concentrations of external LiCl permit lithium to occupy an electronegative pocket in the apical dimer interface, thereby sustaining channel activation. Furthermore, we identified an intersubunit electrostatic bridge adjacent to the pocket that mediates lithium effects on gating. Because LBD dimer pairs appear to be intact in unliganded and preopen, but not desensitized, GluA2 structures (Dürr et al., 2014; Meyerson et al., 2014), the rupture of this bridge might be a key trigger for desensitization. In this sense, lithium acts upon GluA2 as we proposed sodium does for GluK2, serving as a gatekeeper to prevent desensitization (Dawe et al., 2013).

Auxiliary Subunits Rewire the AMPAR Gating Pathway

There is a substantial body of literature describing to what extent TARP and CNIH proteins modulate or, typically, slow AMPAR desensitization and deactivation kinetics (e.g., Priel et al., 2005; Schwenk et al., 2009). Nevertheless, it is presently

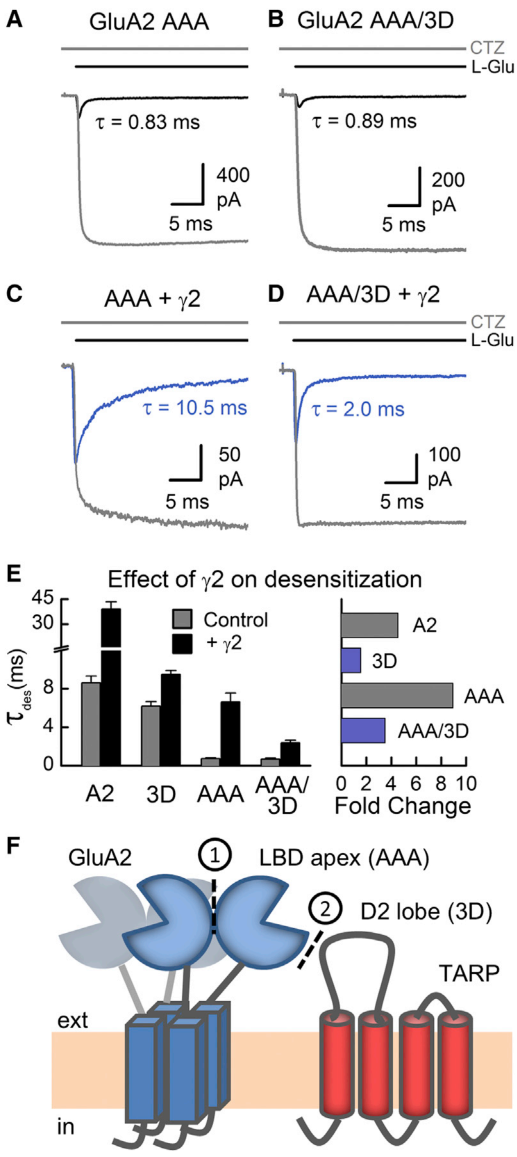

Figure 8. Intra- and Interprotein Interactions Independently Regulate GluA2 Gating

(A–D) Typical current responses of GluA2 AAA (A) (patch number 151005p12), AAA/3D (B) (patch number 151001p11), AAA + γ2 (C) (patch number 140721p3), and AAA/3D + γ2 (D) (patch number 150924p11) mutant receptors to a 250 ms application of 10 mM L-Glu, shown before (black, or blue with γ2) and during (gray) CTZ exposure. Time constants of current decay during desensitization are indicated.

(E) Mean time constants of current decay (τ_{des}, left) for several GluA2 receptors, which were expressed alone (gray bar) or coexpressed with the TARP subunit γ2 (black bar). The ratio of the time constants for each receptor (γ2: no TARP) is also shown, expressed as a fold change (right). Data are mean ± SEM, from the number of independent patch experiments that follows: nine (GluA2), ten (GluA2 + γ2), five (GluA2 3D), seven (GluA2 3D + γ2), seven (GluA2 AAA), eight (GluA2 AAA + γ2), six (GluA2 AAA/3D), and seven (GluA2 AAA/3D + γ2).

(F) Illustration of two distinct LBD regions (apex and D2 lobe) critical for regulating the time course of GluA2 activation, which were disrupted by the AAA and 3D mutations, respectively.

debated whether such effects are mediated primarily through increasing the rate of channel opening, pregating rearrangements of the agonist-binding cleft, or other kinetic transitions. Our observation that the coexpression of auxiliary subunits rescued gating deficits in the GluA2 AAA mutant receptor brings new perspective to how they modulate AMPAR behavior. The Ala mutations were predicted to weaken affinity between individual LBDs, leading dimers to more readily move apart, as is proposed to occur during the structural transition to desensitization (Meyerson et al., 2014; Sun et al., 2002). Because the binding site for CTZ has been well characterized, its rescue of GluA2 AAA could be attributed to the molecule acting as an adhesive in the LBD dimer interface, interfering with the separation of subunits (Sun et al., 2002). In contrast, TARPs and CNIHs are large transmembrane proteins and unlikely to brace the LBD dimer from within, meaning another mechanism should account for their rescue of the AAA mutant.

Cryo-EM experiments have resolved TARP and CNIH proteins situated beside the AMPAR transmembrane domain (TMD), tucked underneath the LBD (Nakagawa et al., 2005; Shanks et al., 2014). More recent assays using antibody labeling of GluA2 peptide arrays have identified several discrete sites to which TARP γ2 may bind, within both the TMD and LBD but also the more distal ATD (Cais et al., 2014). That being said, the LBD appears to be the principle extracellular site where TARPs modulate gating, since removal of the ATD still allows them to promote AMPAR trafficking and modulate decay kinetics (Cais et al., 2014). Specific sites of γ2 interaction identified at the GluA2 LBD include residues that comprise the LBD-TMD linker, segments abutting the agonist-binding cleft, and helices along the D1 dimer interface (Cais et al., 2014). The linker region has been shown to regulate P_{open} of NMDAR channels (Kazi et al., 2014) and could mediate TARP-dependent increases in AMPAR P_{open} (Cho et al., 2007; Tomita et al., 2005). Likewise, more extensive closure of the agonist-binding cleft with γ2 (MacLean et al., 2014) may underlie changes in the relative efficacy of agonists such as KA. Nevertheless, the structural basis for TARP prolongation of channel gating has remained a matter of speculation.

Our identification of a site on the lower, D2 lobe (i.e., the KGK motif) responsible for γ2 modulation of GluA2 deactivation and desensitization kinetics sheds new light on the functional interaction between TARP and AMPAR subunits. Specifically, we propose that TARP auxiliary subunits provide external stabilization at the base of the LBD dimer, interfering with the turning apart and/or separation of receptor subunits that characterizes desensitization (Meyerson et al., 2014; Dürr et al., 2014). The low, outward-facing orientation of the KGK motif is also consistent with the predicted location of TARP subunits in native AMPAR complexes (Nakagawa et al., 2005). Moreover, the continued importance of the KGK residues for γ2 coexpression to rescue gating of GluA2 AAA receptors demonstrates that interprotein interactions relayed through the basal D2 lobe operate independently of the electrostatic interactions at the LBD apex. Given that the KGK motif did not affect TARP modulation of agonist efficacy or polyamine block, it is likely that several other discrete interactions are required to achieve the full set of TARP effects. As such, auxiliary proteins add additional branches to

the intrinsic gating machinery of pore-forming AMPAR subunits, coordinating receptor activation through distinct structural pathways.

EXPERIMENTAL PROCEDURES

Molecular Biology, Electrophysiology, and Surface Expression

HEK293T cells were used to recombinantly express KAR or AMPAR subunits for outside-out patch recordings and surface-expression assays. For AMPARs, the Q/R unedited, flip variant of subunits was used, and residue numbering includes the signal peptide. Mutant receptors were generated using site-directed mutagenesis. Auxiliary subunits and AMPARs were coexpressed at a 2:1 cDNA ratio. External and internal recording solutions typically contained 150 mM XCl (X = alkali metal), 5 mM HEPES, 0.1 mM $CaCl_2$, 0.1 mM $MgCl_2$, and 2% phenol red at pH 7.4; and 115 mM NaCl, 10 mM NaF, 5 mM HEPES, 5 mM Na_4BAPTA, 0.5 mM $CaCl_2$, 1 mM $MgCl_2$, and 10 mM Na_2ATP at pH 7.4, respectively. L-Glu was typically applied at 10 mM and CTZ at 100 μM. Agonist solutions were applied using a piezo-stack-driven perfusion system, and measured solution exchange time was under 400 μs. The recording, acquisition, and analysis of electrophysiological data are detailed in Supplemental Experimental Procedures. Membrane trafficking was assessed from the fluorescence emitted by an ecliptic, pH-sensitive superfolder GFP genetically fused to the extracellular amino terminal of AMPARs, as described previously for KARs (Dawe et al., 2013). Additional details are described in Supplemental Experimental Procedures.

MD Simulations

The GluA2 flip (PDB: 2UXA; Greger et al., 2006) and K759M/T765K LBD dimers were used for constructing models for MD simulations. Proteins were solvated, ions were introduced, and mutations were imposed prior to simulation. MD simulations were performed using Gromacs 4.6 (Hess et al., 2008) with the OPLS all-atom force field (Jorgensen et al., 1996; Kaminski et al., 2001). Periodic boundary conditions were employed, while electrostatic interactions and bonds were accounted for as described previously (Dawe et al., 2013). Simulations of 100 ns were performed in the NPT ensemble at 300 K and 1 bar pressure using the Berendsen thermostat and barostat, respectively (Berendsen et al., 1984). Two to four repeats for each wild-type or mutant dimer were produced. Analyses were performed using VMD (Humphrey et al., 1996) and Gromacs (Hess et al., 2008). Additional details are described in Supplemental Experimental Procedures.

X-Ray Crystallography

The GluA2 (flip) K759M/T765K LBD construct was generated from the wild-type GluA2 LBD (provided by Ingo Greger) using the QuikChange protocol (Stratagene). Induction and expression (1 mM IPTG, 20 hr at 24°C) were followed by protoplast formation and freeze-thaw lysis. Purification of the resulting supernatant on nickel-affinity and HiTrap-Q columns was performed as described previously (Nayeem et al., 2011). Crystals were grown as described in Supplemental Experimental Procedures. Diffraction data were collected at 100 K on Diamond beamline I03 at an energy of 12,700 eV (Pilatus3 6M detector). Data processing was performed using either XDS/XSCALE (lithium form) or XDS/AIMLESS (zinc form). Molecular replacement was performed in PHASER, and refinement was performed using a combination of REFMAC5 (Murshudov et al., 1997) and PHENIX.REFINE (Adams et al., 2002). For the zinc structure, PHASER was used for SAD-MR to locate the five zinc ions, and for map generation, either map sharpening (REFMAC5) or feature-enhanced maps (PHENIX.REFINE) were used. TLS groups were identified using the TLSMD server (Painter and Merritt, 2006). In all cases, model visualization and manipulation were done using COOT (Emsley et al., 2010), and figures were generated using CCP4MG (McNicholas et al., 2011). Additional details are described in Supplemental Experimental Procedures.

Statistical Methods

Results are expressed as mean ± SEM. Statistical analyses of sample means were performed using two-tailed paired or two-sample (assuming unequal variance) t tests. $p < 0.05$ was considered to be statistically significant.

ACCESSION NUMBERS

Model coordinates and diffraction data for the GluA2 K759M/T765K structures have been deposited in the Protein Data Bank under ID codes PDB: 5FTH (zinc form) and 5FTI (lithium form).

SUPPLEMENTAL INFORMATION

Supplemental Information includes Supplemental Experimental Procedures, seven figures, three tables, and four movies and can be found with this article online at http://dx.doi.org/10.1016/j.neuron.2016.01.038.

AUTHOR CONTRIBUTIONS

Conceptualization, G.B.D., M.R.P.A., and D.B. Investigation and Analysis–Electrophysiology, G.B.D.; Investigation and Analysis–Molecular Biology, G.B.D. and M.R.P.A.; Investigation and Analysis–Surface Expression, M.R.P.A.; Investigation and Analysis–MD Simulations, M.M.; Investigation and Analysis–Crystallography, N.N. and T.G. Writing, G.B.D. and D.B. Review and Editing, all authors.

ACKNOWLEDGMENTS

This work was supported by operating grants from the Canadian Institutes of Health Research (FRN: 82804, D.B.), the Leverhulme Trust (RPG-059, P.C.B. and M.M.), and the Medical Research Council (MR/M0004331, P.C.B.). G.B.D. is supported by a Canada Graduate Scholarship (CGS-D) from the Natural Sciences and Engineering Research Council of Canada. M.M. held a postdoctoral fellowship from the Alfred Benzon Foundation. D.B. was a Canada Research Chair. We thank the Advanced Research Computing (ARC) facility, the Blue Joule facility at the Hartree Centre, the National Service for Computational Chemistry Software, the IRIDIS High Performance Computing facility, and the HECTOR and ARCHER UK National Supercomputing Services for computer time through the EPSRC-funded HECBioSim consortium (www.hecbiosim.ac.uk), as well as the Diamond Synchrotron for beam time (provided through the Manchester/Liverpool BBSRC BAG). We also thank Jennifer Flemming for collecting the P2 21 21 dataset at Diamond and Dr. Gergely Lukacs for use of his TIRF microscope.

Received: July 30, 2015
Revised: December 1, 2015
Accepted: January 13, 2016
Published: February 25, 2016

REFERENCES

Adams, P.D., Grosse-Kunstleve, R.W., Hung, L.W., Ioerger, T.R., McCoy, A.J., Moriarty, N.W., Read, R.J., Sacchettini, J.C., Sauter, N.K., and Terwilliger, T.C. (2002). PHENIX: building new software for automated crystallographic structure determination. Acta Crystallogr. D Biol. Crystallogr. *58*, 1948–1954.

Armstrong, N., and Gouaux, E. (2000). Mechanisms for activation and antagonism of an AMPA-sensitive glutamate receptor: crystal structures of the GluR2 ligand binding core. Neuron *28*, 165–181.

Assaf, Z., Larsen, A.P., Venskutonytė, R., Han, L., Abrahamsen, B., Nielsen, B., Gajhede, M., Kastrup, J.S., Jensen, A.A., Pickering, D.S., et al. (2013). Chemoenzymatic synthesis of new 2,4-syn-functionalized (S)-glutamate analogues and structure-activity relationship studies at ionotropic glutamate receptors and excitatory amino acid transporters. J. Med. Chem. *56*, 1614–1628.

Berendsen, H.J.C., Postma, J.P.M., Vangunsteren, W.F., Dinola, A., and Haak, J.R. (1984). Molecular-dynamics with coupling to an external bath. J. Chem. Phys. *81*, 3684–3690.

Bowie, D. (2002). External anions and cations distinguish between AMPA and kainate receptor gating mechanisms. J. Physiol. *539*, 725–733.

Bowie, D. (2010). Ion-dependent gating of kainate receptors. J. Physiol. *588*, 67–81.

Cais, O., Herguedas, B., Krol, K., Cull-Candy, S.G., Farrant, M., and Greger, I.H. (2014). Mapping the interaction sites between AMPA receptors and TARPs reveals a role for the receptor N-terminal domain in channel gating. Cell Rep. *9*, 728–740.

Catterall, W.A., Hulme, J.T., Jiang, X., and Few, W.P. (2006). Regulation of sodium and calcium channels by signaling complexes. J. Recept. Signal Transduct. Res. *26*, 577–598.

Chen, L., El-Husseini, A., Tomita, S., Bredt, D.S., and Nicoll, R.A. (2003). Stargazin differentially controls the trafficking of alpha-amino-3-hydroxyl-5-methyl-4-isoxazolepropionate and kainate receptors. Mol. Pharmacol. *64*, 703–706.

Cho, C.H., St-Gelais, F., Zhang, W., Tomita, S., and Howe, J.R. (2007). Two families of TARP isoforms that have distinct effects on the kinetic properties of AMPA receptors and synaptic currents. Neuron *55*, 890–904.

Constals, A., Penn, A.C., Compans, B., Toulmé, E., Phillipat, A., Marais, S., Retailleau, N., Hafner, A.S., Coussen, F., Hosy, E., and Choquet, D. (2015). Glutamate-induced AMPA receptor desensitization increases their mobility and modulates short-term plasticity through unbinding from Stargazin. Neuron *85*, 787–803.

Dawe, G.B., Musgaard, M., Andrews, E.D., Daniels, B.A., Aurousseau, M.R., Biggin, P.C., and Bowie, D. (2013). Defining the structural relationship between kainate-receptor deactivation and desensitization. Nat. Struct. Mol. Biol. *20*, 1054–1061.

Dawe, G.B., Aurousseau, M.R., Daniels, B.A., and Bowie, D. (2015). Retour aux sources: defining the structural basis of glutamate receptor activation. J. Physiol. *593*, 97–110.

Dingledine, R., Borges, K., Bowie, D., and Traynelis, S.F. (1999). The glutamate receptor ion channels. Pharmacol. Rev. *51*, 7–61.

Dürr, K.L., Chen, L., Stein, R.A., De Zorzi, R., Folea, I.M., Walz, T., Mchaourab, H.S., and Gouaux, E. (2014). Structure and dynamics of AMPA receptor GluA2 in resting, pre-open, and desensitized states. Cell *158*, 778–792.

Emsley, P., Lohkamp, B., Scott, W.G., and Cowtan, K. (2010). Features and development of Coot. Acta Crystallogr. D Biol. Crystallogr. *66*, 486–501.

Gan, Q., Salussolia, C.L., and Wollmuth, L.P. (2015). Assembly of AMPA receptors: mechanisms and regulation. J. Physiol. *593*, 39–48.

Gebhardt, C., and Cull-Candy, S.G. (2010). Lithium acts as a potentiator of AMPAR currents in hippocampal CA1 cells by selectively increasing channel open probability. J. Physiol. *588*, 3933–3941.

Green, T., and Nayeem, N. (2015). The multifaceted subunit interfaces of ionotropic glutamate receptors. J. Physiol. *593*, 73–81.

Greger, I.H., Akamine, P., Khatri, L., and Ziff, E.B. (2006). Developmentally regulated, combinatorial RNA processing modulates AMPA receptor biogenesis. Neuron *51*, 85–97.

Greger, I.H., Ziff, E.B., and Penn, A.C. (2007). Molecular determinants of AMPA receptor subunit assembly. Trends Neurosci. *30*, 407–416.

Haering, S.C., Tapken, D., Pahl, S., and Hollmann, M. (2014). Auxiliary subunits: shepherding AMPA receptors to the plasma membrane. Membranes (Basel) *4*, 469–490.

Hastie, P., Ulbrich, M.H., Wang, H.L., Arant, R.J., Lau, A.G., Zhang, Z., Isacoff, E.Y., and Chen, L. (2013). AMPA receptor/TARP stoichiometry visualized by single-molecule subunit counting. Proc. Natl. Acad. Sci. USA *110*, 5163–5168.

Herring, B.E., Shi, Y., Suh, Y.H., Zheng, C.Y., Blankenship, S.M., Roche, K.W., and Nicoll, R.A. (2013). Cornichon proteins determine the subunit composition of synaptic AMPA receptors. Neuron *77*, 1083–1096.

Hess, B., Kutzner, C., van der Spoel, D., and Lindahl, E. (2008). GROMACS 4: algorithms for highly efficient, load-balanced, and scalable molecular simulation. J. Chem. Theory Comput. *4*, 435–447.

Horning, M.S., and Mayer, M.L. (2004). Regulation of AMPA receptor gating by ligand binding core dimers. Neuron *41*, 379–388.

Howe, J.R. (2015). Modulation of non-NMDA receptor gating by auxiliary subunits. J. Physiol. *593*, 61–72.

Huettner, J.E. (2015). Glutamate receptor pores. J. Physiol. *593*, 49–59.

Humphrey, W., Dalke, A., and Schulten, K. (1996). VMD: visual molecular dynamics. J. Mol. Graph. *14*, 33–38.

Jackson, A.C., and Nicoll, R.A. (2011). The expanding social network of ionotropic glutamate receptors: TARPs and other transmembrane auxiliary subunits. Neuron *70*, 178–199.

Jorgensen, W.L., Maxwell, D.S., and Tirado-Rives, J. (1996). Development and testing of the OPLS all-atom force field on conformational energetics and properties of organic liquids. J. Am. Chem. Soc. *118*, 11225–11236.

Kalashnikova, E., Lorca, R.A., Kaur, I., Barisone, G.A., Li, B., Ishimaru, T., Trimmer, J.S., Mohapatra, D.P., and Díaz, E. (2010). SynDIG1: an activity-regulated, AMPA- receptor-interacting transmembrane protein that regulates excitatory synapse development. Neuron *65*, 80–93.

Kaminski, G.A., Friesner, R.A., Tirado-Rives, J., and Jorgensen, W.L. (2001). Evaluation and reparametrization of the OPLS-AA force field for proteins via comparison with accurate quantum chemical calculations on peptides. J. Phys. Chem. B *105*, 6474–6487.

Karkanias, N.B., and Papke, R.L. (1999). Lithium modulates desensitization of the glutamate receptor subtype gluR3 in Xenopus oocytes. Neurosci. Lett. *277*, 153–156.

Kazi, R., Dai, J., Sweeney, C., Zhou, H.X., and Wollmuth, L.P. (2014). Mechanical coupling maintains the fidelity of NMDA receptor-mediated currents. Nat. Neurosci. *17*, 914–922.

MacLean, D.M., Ramaswamy, S.S., Du, M., Howe, J.R., and Jayaraman, V. (2014). Stargazin promotes closure of the AMPA receptor ligand-binding domain. J. Gen. Physiol. *144*, 503–512.

McNicholas, S., Potterton, E., Wilson, K.S., and Noble, M.E. (2011). Presenting your structures: the CCP4mg molecular-graphics software. Acta Crystallogr. D Biol. Crystallogr. *67*, 386–394.

Meyerson, J.R., Kumar, J., Chittori, S., Rao, P., Pierson, J., Bartesaghi, A., Mayer, M.L., and Subramaniam, S. (2014). Structural mechanism of glutamate receptor activation and desensitization. Nature *514*, 328–334.

Morimoto-Tomita, M., Zhang, W., Straub, C., Cho, C.H., Kim, K.S., Howe, J.R., and Tomita, S. (2009). Autoinactivation of neuronal AMPA receptors via glutamate-regulated TARP interaction. Neuron *61*, 101–112.

Murshudov, G.N., Vagin, A.A., and Dodson, E.J. (1997). Refinement of macromolecular structures by the maximum-likelihood method. Acta Crystallogr. D Biol. Crystallogr. *53*, 240–255.

Nakagawa, T., Cheng, Y., Ramm, E., Sheng, M., and Walz, T. (2005). Structure and different conformational states of native AMPA receptor complexes. Nature *433*, 545–549.

Nayeem, N., Mayans, O., and Green, T. (2011). Conformational flexibility of the ligand-binding domain dimer in kainate receptor gating and desensitization. J. Neurosci. *31*, 2916–2924.

Painter, J., and Merritt, E.A. (2006). Optimal description of a protein structure in terms of multiple groups undergoing TLS motion. Acta Crystallogr. D Biol. Crystallogr. *62*, 439–450.

Plested, A.J., Vijayan, R., Biggin, P.C., and Mayer, M.L. (2008). Molecular basis of kainate receptor modulation by sodium. Neuron *58*, 720–735.

Priel, A., Kolleker, A., Ayalon, G., Gillor, M., Osten, P., and Stern-Bach, Y. (2005). Stargazin reduces desensitization and slows deactivation of the AMPA-type glutamate receptors. J. Neurosci. *25*, 2682–2686.

Schwenk, J., Harmel, N., Zolles, G., Bildl, W., Kulik, A., Heimrich, B., Chisaka, O., Jonas, P., Schulte, U., Fakler, B., and Klöcker, N. (2009). Functional proteomics identify cornichon proteins as auxiliary subunits of AMPA receptors. Science *323*, 1313–1319.

Severus, W.E., Kleindienst, N., Seemüller, F., Frangou, S., Möller, H.J., and Greil, W. (2008). What is the optimal serum lithium level in the long-term treatment of bipolar disorder—a review? Bipolar Disord. *10*, 231–237.

Shanks, N.F., Cais, O., Maruo, T., Savas, J.N., Zaika, E.I., Azumaya, C.M., Yates, J.R., 3rd, Greger, I., and Nakagawa, T. (2014). Molecular dissection of the interaction between the AMPA receptor and cornichon homolog-3. J. Neurosci. *34*, 12104–12120.

Sobolevsky, A.I., Rosconi, M.P., and Gouaux, E. (2009). X-ray structure, symmetry and mechanism of an AMPA-subtype glutamate receptor. Nature *462*, 745–756.

Soto, D., Coombs, I.D., Kelly, L., Farrant, M., and Cull-Candy, S.G. (2007). Stargazin attenuates intracellular polyamine block of calcium-permeable AMPA receptors. Nat. Neurosci. *10*, 1260–1267.

Sun, Y., Olson, R., Horning, M., Armstrong, N., Mayer, M., and Gouaux, E. (2002). Mechanism of glutamate receptor desensitization. Nature *417*, 245–253.

Tomita, S., Chen, L., Kawasaki, Y., Petralia, R.S., Wenthold, R.J., Nicoll, R.A., and Bredt, D.S. (2003). Functional studies and distribution define a family of transmembrane AMPA receptor regulatory proteins. J. Cell Biol. *161*, 805–816.

Tomita, S., Adesnik, H., Sekiguchi, M., Zhang, W., Wada, K., Howe, J.R., Nicoll, R.A., and Bredt, D.S. (2005). Stargazin modulates AMPA receptor gating and trafficking by distinct domains. Nature *435*, 1052–1058.

Trimmer, J.S. (2015). Subcellular localization of K+ channels in mammalian brain neurons: remarkable precision in the midst of extraordinary complexity. Neuron *85*, 238–256.

Turetsky, D., Garringer, E., and Patneau, D.K. (2005). Stargazin modulates native AMPA receptor functional properties by two distinct mechanisms. J. Neurosci. *25*, 7438–7448.

von Engelhardt, J., Mack, V., Sprengel, R., Kavenstock, N., Li, K.W., Stern-Bach, Y., Smit, A.B., Seeburg, P.H., and Monyer, H. (2010). CKAMP44: a brain-specific protein attenuating short-term synaptic plasticity in the dentate gyrus. Science *327*, 1518–1522.

Wong, A.Y., Fay, A.M., and Bowie, D. (2006). External ions are coactivators of kainate receptors. J. Neurosci. *26*, 5750–5755.

Zhang, W., Devi, S.P., Tomita, S., and Howe, J.R. (2014). Auxiliary proteins promote modal gating of AMPA- and kainate-type glutamate receptors. Eur. J. Neurosci. *39*, 1138–1147.

Neuron, Volume *89*

Supplemental Information

Distinct Structural Pathways Coordinate the Activation of AMPA Receptor-Auxiliary Subunit Complexes

G. Brent Dawe, Maria Musgaard, Mark R.P. Aurousseau, Naushaba Nayeem, Tim Green, Philip C. Biggin, and Derek Bowie

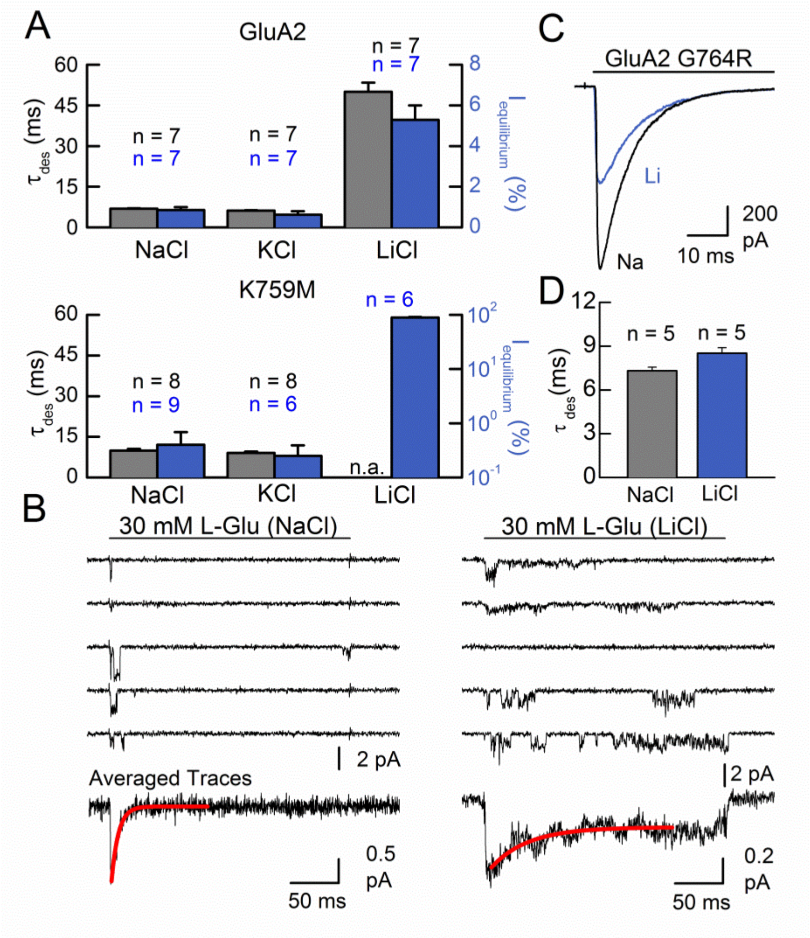

Figure S1 Functional properties of GluA2 receptors in different external ionic conditions. Supplemental data associated with Figure 1.

Since cation substitution experiments at GluA1 AMPARs do not affect the time course of channel activation (Bowie, 2002), we have explored whether GluA2 AMPARs are sensitive to other cation species beside lithium (i.e. potassium), and whether any structural features account for the difference in ion modulation between AMPAR subunits. One difference between GluA1 and GluA2 AMPARs is the R/G RNA editing site, located at the apex of the LBD, adjacent to the electronegative pocket on the same and opposing (across the dimer interface) subunits. This residue is edited (i.e. Gly) in GluA2, but unedited (i.e. Arg) in GluA1 (Lomeli et al., 1994). To determine whether the editing state of the R/G site affects lithium modulation of GluA2, we measured desensitization kinetics in the GluA2 G764R mutant.

(A) Current decay time constants (left, grey) and equilibrium to peak current ratios (right, blue) for wildtype GluA2 (top) and K759M (bottom) mutant receptors in the presence of different

cation species. Data are mean ± SEM, from the number of independent patch experiments indicated.

(B) Typical GluA2 unitary current events elicited by 30 mM L-Glu in NaCl (left) (Patch #140128p7) and LiCl (right) (Patch # 140121p10) external solutions. Below is an average of several individual sweeps, including a single, exponential function fit (red) of the current decay.

(C) Typical current responses elicited by 10 mM L-Glu on GluA2 G764R mutant receptors in external NaCl and LiCl (Patch # 150203p3).

(D) Average current decay time constants for GluA2 G764R receptors. Consistent with the behavior of GluA1 receptors, the slowing of wildtype GluA2 receptor decay kinetics observed with lithium was almost entirely eliminated in G764R mutant receptors, which exhibited desensitization time constants of 7.3 ± 0.2 ms (n = 5) in NaCl and 8.5 ± 0.4 ms (n = 5) in LiCl. Data are mean ± SEM, from the number of independent patch experiments indicated.

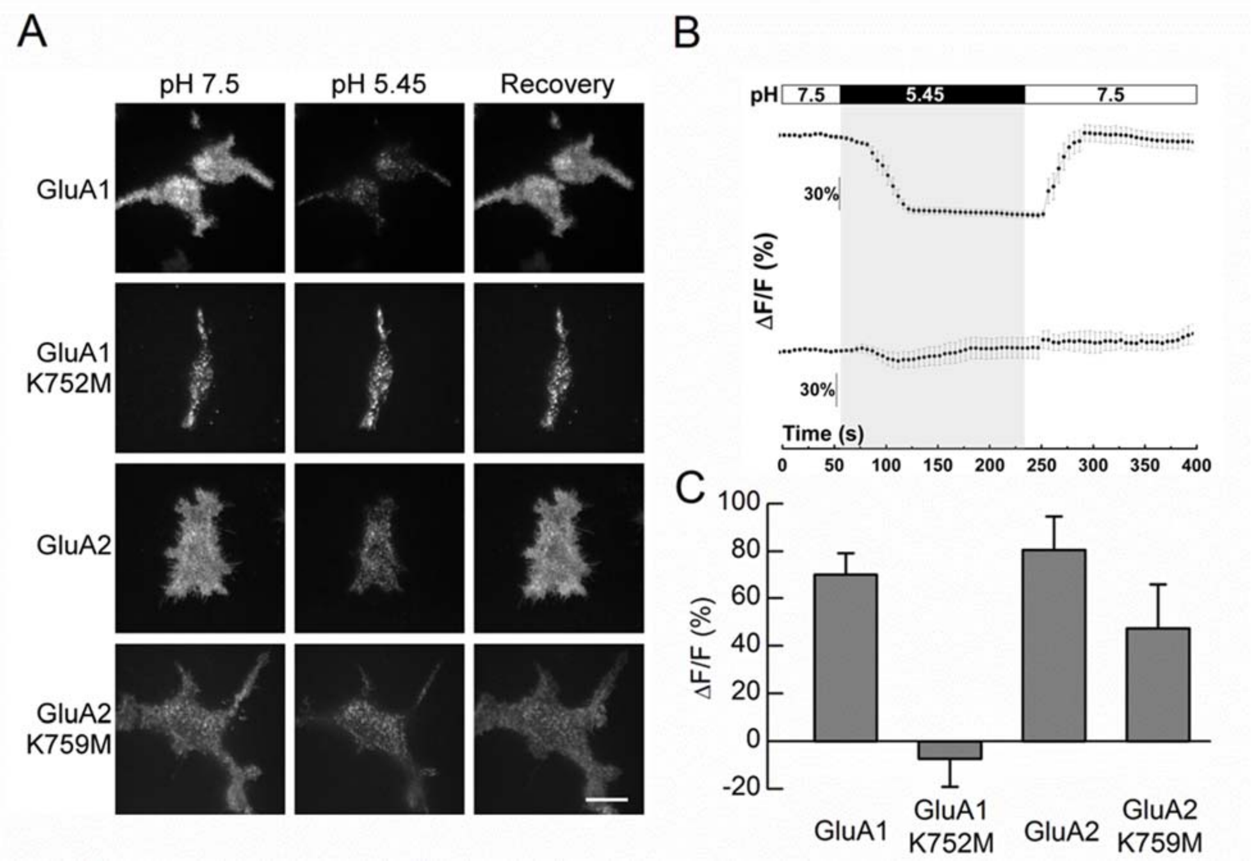

Figure S2 Surface expression of AMPAR subunits harboring an equivalent Lys to Met mutation at the electronegative pocket. Supplemental data associated with Figure 2.

(A) TIRF images of HEK293T cells transfected with GFP-tagged wildtype and mutant GluA1 and GluA2 receptors exhibit reversible attenuation of the fluorescence signal between pH 7.5 and 5.45 when subunits are expressed on the plasma membrane (scale bar = 20 µm).

(B) Individual, time-resolved fluorescence profiles for single cells expressing either wildtype GluA1 (top) or the K752M mutant (bottom).

(C) Bar graph tabulating the change in fluorescent signal observed for cells expressing wildtype and mutant AMPARs. Data are mean ± SEM, from five (GluA1 K752M), ten (wildtype GluA1), thirteen (GluA2 K759M), or eighteen (wildtype GluA2) individual cell imaging experiments for each receptor.

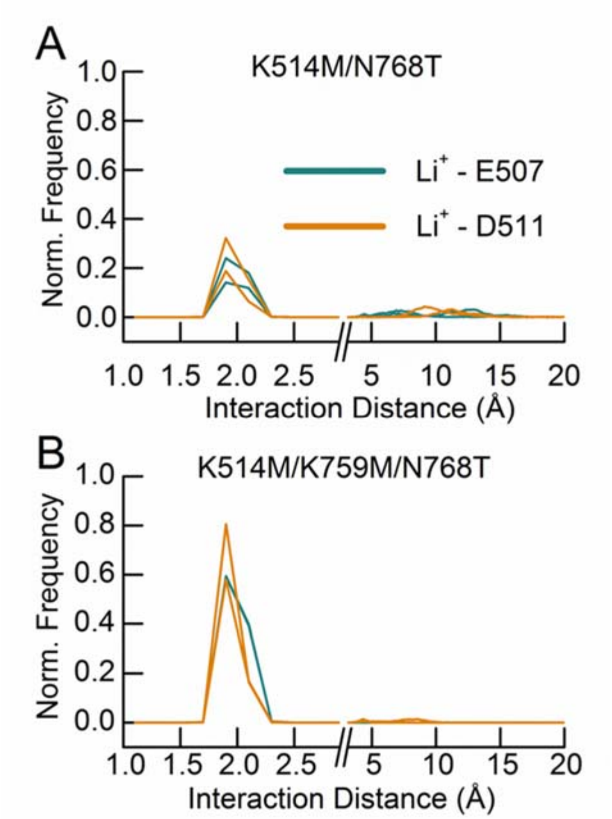

Figure S3 Lithium binding properties of the GluA2 K514M/N768T mutant receptor. Supplemental data associated with Figure 3.

(A and B) Data from MD simulations reporting the interaction distance between residue Glu507 or Asp511and the nearest lithium ion, for K514M/N768T (A) and K514M/K759M/N768T (B) mutant GluA2 receptors. Data from the triple mutant (i.e. K514M/N768T + K759M) was included to take advantage of the increased frequency of lithium binding measured in prior simulations following addition of the K759M mutation (Figure 1). Distance was measured from the sidechain oxygen atom closest to lithium on the residues indicated. Frequency is normalized (bin size = 0.2 Å, cumulative frequency = 1.0) and averaged from two simulation repeats of 100 ns for each receptor. Values for each chain (A and B) in the LBD dimer are shown.

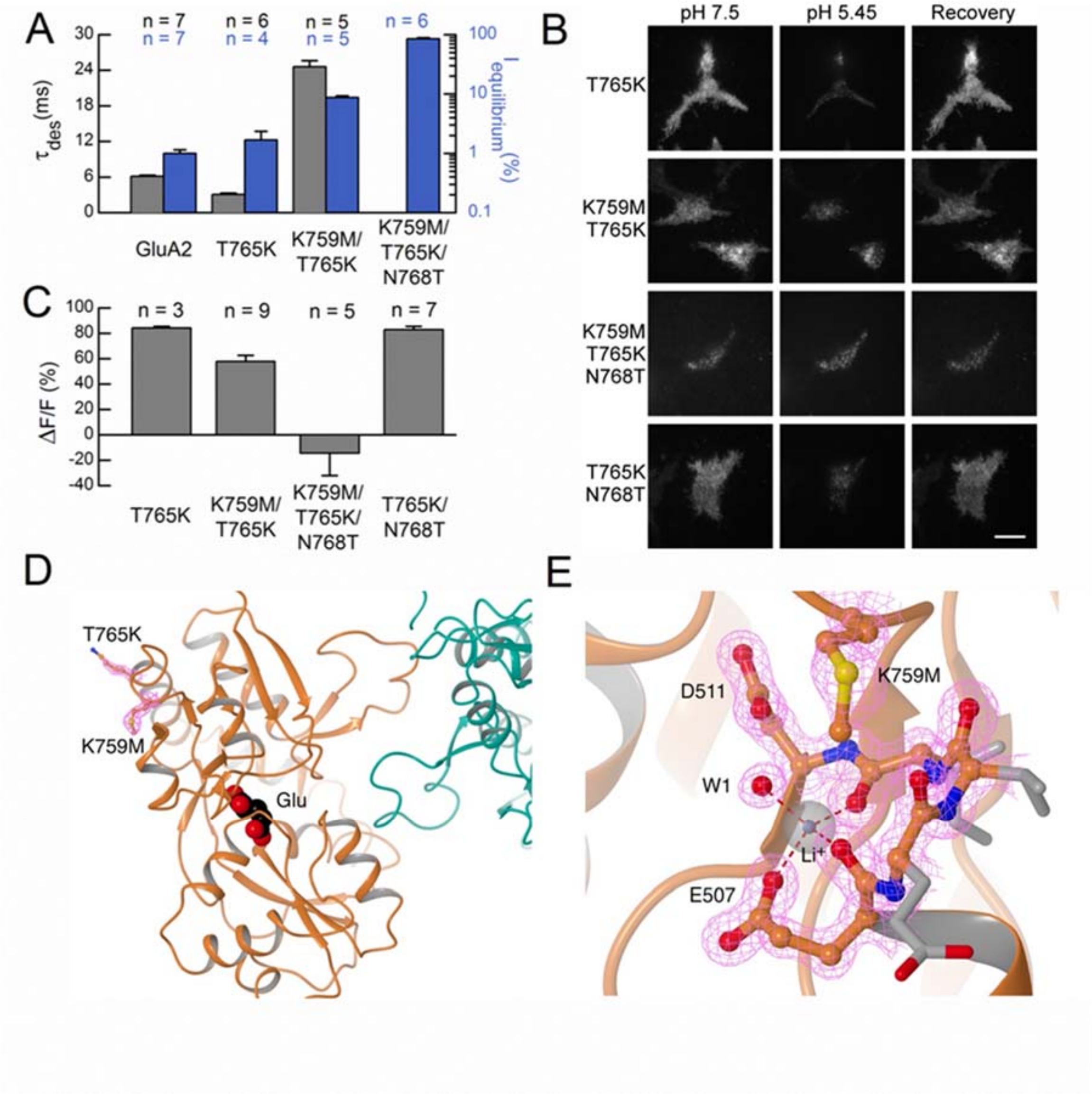

Figure S4 Structure, function, and surface expression of the GluA2 T765K mutant series of receptors. Supplemental data associated with Figure 4.

Our previous work has established that GluK2 KAR desensitization is abolished by the D776K mutation, which acts as a cross-dimer electrostatic tether onto the cation binding pocket (Dawe et al., 2013; Nayeem et al., 2011; Nayeem et al., 2009). We created a series of mutants incorporating the equivalent mutation in GluA2, namely T765K, in the hopes of achieving a similar tether between GluA2 subunits. Perhaps because Lys759 interferes with the tethering of the mutant lysine at the electronegative pocket of GluA2 (Figure 1), the addition of the K759M mutation was required atop T765K to prolong the time course of current responses. As such, we focussed our analysis on GluA2 K759M/T765K, for which we were able to crystalize a

crosslinked LBD dimer, and also GluA2 K759M/T765K/N768T (MKT), which showed little, if any, detectable current decay over 250 ms L-Glu applications. Nevertheless, kinetic analysis of GluA2 MKT was made difficult due to its greatly diminished surface expression.

(A) Time constants of current decay (left, grey) and equilibrium to peak current ratio (right, blue) for the GluA2 T765K series of mutants. Data are mean ± SEM, from the number of independent patch experiments indicated.

(B) TIRF images of HEK293T cells transfected with wildtype GluA2 or one of several mutant receptors possessing Lys at the 765 position. Reversible attenuation of the GFP fluorescence signal occurs between pH 7.5 and 5.45 when subunits are expressed on the plasma membrane (scale bar = 20 μm).

(C) Bar graph tabulating the change in fluorescent signal observed for cells expressing wildtype and mutant AMPARs. Data are mean ± SEM, from the number of independent patch experiments indicated.

(D) View of protomer A (orange), with the two mutated residues shown with associated electron density ($|2F_{obs} - F_{calc}|\alpha_{calc}$; pink mesh, contoured at 1.5σ). The L-Glu ligand is shown in black space-fill. Part of protomer B (teal) can be seen to the right, highlighting the absence of the biological dimer from this crystal form.

(E) Closer view of protomer A (orange) from approximately the viewpoint shown in Figure 4. The modeled lithium ion (gray sphere) is shown, along with an interacting water (W1) and other atoms within the electronegative pocket. Electron density ($|2F_{obs} - F_{calc}|\alpha_{calc}$) is shown contoured at 2σ around the displayed atoms (pink mesh) with the exception of the lithium ion, where it is displayed at 1σ (gray mesh). Contacts between the lithium ion and other atoms are shown as dashed lines.

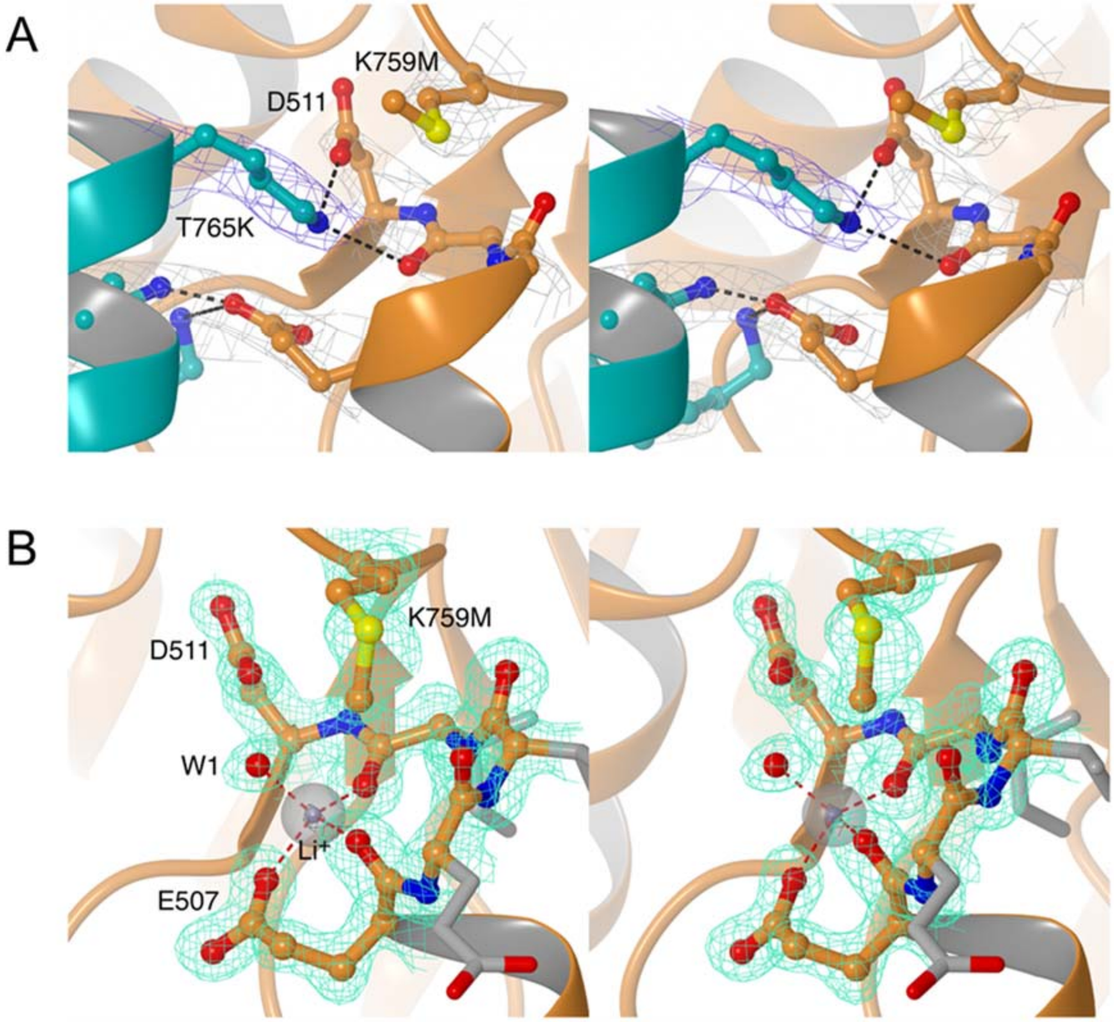

Figure S5 Stereo views of composite omit maps of the GluA2 K759M/T765K LBD, generated using PHENIX. Supplemental data associated with Figure 4.

(A) View of the mutant T765K residue on protomer B of the zinc crystal form, interacting with residues in the electronegative pocket of protomer A. Residue and density labelling is maintained as for Figure 4, with contours displayed at 1.2 σ.

(B) View of the electronegative binding pocket on protomer A of the lithium crystal form. Residue and density labelling is maintained as for Supplemental Figure 4, with contours displayed at 1.5 σ (or 0.8 σ around the lithium ion).

			Helix D		Helix J
NP_113796.1	GluA1	494	TITLVREEVIDFSKP	754	SALRNPVNLAVLKL
NP_058957.1	GluA2	501	TITLVREEVIDFSKP	761	SSLGTPVNLAVLKL
NP_116785.2	GluA3	504	TITLVREEVIDFSKP	766	SALGTPVNLAVLKL
NP_058959.2	GluA4	502	TITLVREEVIDFSKP	762	SSLRTPVNLAVLKL
NP_058937.1	GluK1	533	TITYVREKVIDFSKP	787	SPYRDKITIAILQL
NP_062182.1	GluK2	518	AITYVREKVIDFSKP	772	SPYRDKITIAILQL
NP_852038.2	GluK3	520	TITHVREKAIDFSKP	773	SPYRDKITIAILQL
NP_036704.1	GluK4	502	TITAEREKVIDFSKP	757	SVFRDEFDLAILQL
NP_113696.1	GluK5	501	TITAEREKVIDFSKP	756	SPFRDEITLAILQL
NP_058706.1	GluN1	518	TINNERAQYIEFSKP	766	SPWKQNVSLSILKS
NP_036705.3	GluN2A	513	TINEERSEVVDFSVP	769	SPWKRQIDLALLQF
NP_036706.1	GluN2B	514	TINEERSEVVDFSVP	770	SGWKRQVDLAILQL
NP_036707.3	GluN2C	524	TINEERSEIIDFSVP	780	SHWKRAIDLALLQL
NP_073634.1	GluN2D	538	TINEERSEIVDFSVP	794	SRWKRPIDLALLQF
NP_612555.1	GluN3A	633	SINTARSQVIDFTSP	881	SPLTSNISELISQY
NP_579842.2	GluN3B	524	SINSARSQVVDFTSP	781	SPLTSNLSEFISRY

Figure S6 Amino acid sequence alignment of iGluRs at the apical LBD dimer interface. Supplemental data associated with Figure 5.

Amino acid sequences of iGluR subunits from *R. norvegicus*, aligned over two segments of the LBD. The NCBI accession code is shown at left. For AMPAR subunits, the flip isoform was selected. Residues participating in cross-dimer electrostatic interactions at the apex of the GluA2 LBD are highlighted magenta. When these residues are not conserved with the equivalent AMPAR residue they are highlighted cyan. The conserved Phe residue at position 512 could not be mutated alongside other resides in the GluA2 AAA mutant, since its contribution to the electrostatic network is from a backbone oxygen atom. Nevertheless, both residues across the dimer interface that would be expected to interact with Phe512, namely Lys514 and Asn768, were truncated.

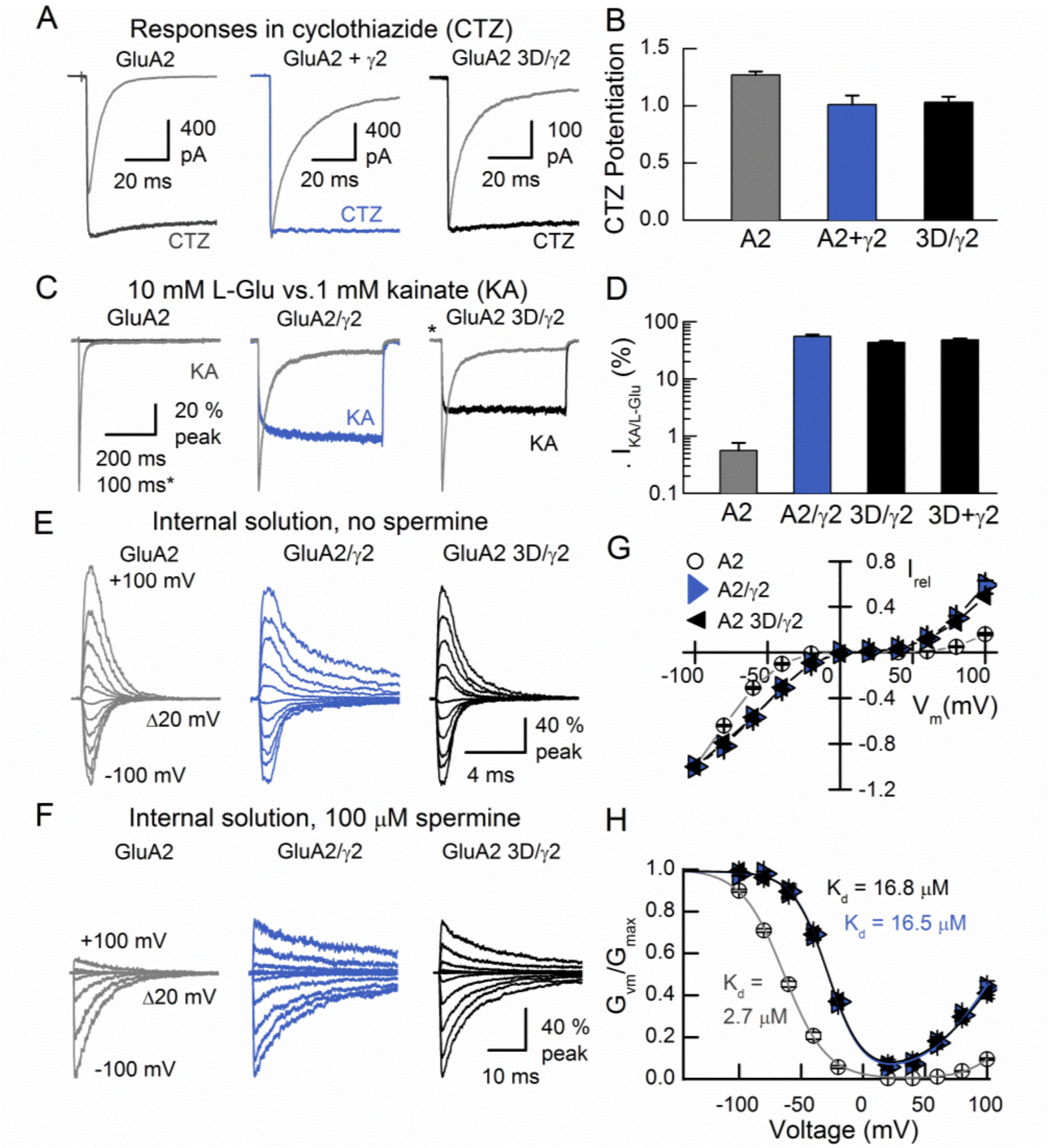

Figure S7 The GluA2 3D mutation does not attenuate TARP γ2 modulation of apparent agonist efficacy or channel block by spermine. Supplemental data associated with Figure 7.

(A) Typical current responses to 10 mM L-Glu before (grey) and during CTZ exposure for wildtype GluA2 (Patch # 130217p8, dark grey), as well as co-expressed GluA2 + γ2 (Patch # 150305p7, blue) and the GluA2 3D/γ2 (Patch # 150911p1, black) AMPAR-TARP fusion protein.

(B) Mean CTZ potentiation of the receptors described in panel A. Values for GluA2 and GluA2 + γ2 correspond to those reported in Figures 5 and 6. Data are mean ± SEM, from eleven (GluA2), five (GluA2 + γ2), or six (GluA2 3D/γ2) independent patch experiments.

(C) Scaled current responses to 10 mM L-Glu (grey) and 1 mM KA for wildtype GluA2 (Patch # 150317p3, dark grey), as well as GluA2/γ2 (Patch # 150316p10, blue) and GluA2 3D/γ2 (Patch # 150511p6, black) AMPAR-TARP fusion proteins.

(D) Mean 1mM KA response, as a percentage of the peak current yielded by 10 mM L-Glu, for the receptors in panel C, as well as GluA2 3D + γ2 (TARP co-expressed). Data are mean ± SEM, from five (GluA2 and GluA2 3D/γ2), six (GluA2/γ2) and seven (GluA2 3D + γ2) independent patch experiments.

(E) Scaled current responses to 10 mM L-Glu at a range of membrane potentials (-100 to +100 mV, Δ20 mV) for wildtype GluA2 (Patch # 150716p10, grey), as well as GluA2/γ2 (Patch # 150723p11, blue) and GluA2 3D/γ2 (Patch # 150911p6, black) AMPAR-TARP fusion proteins.

(F) Scaled current responses to 10 mM L-Glu at a range of membrane potentials (-100 to +100 mV, Δ20 mV) with 100 μM spermine added to the internal recording solution for wildtype GluA2 (Patch # 150525p5, grey), as well as GluA2/γ2 (Patch # 150528p11, blue) and GluA2 3D/γ2 (Patch # 150910p7, black) AMPAR-TARP fusion proteins.

(G) Current-voltage plots with 100 μM internal spermine for wildtype GluA2 (white circles), as well as GluA2/γ2 (blue triangles) and GluA2 3D/γ2 (black triangles) AMPAR-TARP fusion proteins. Currents were normalized to the response at -100 mV (I_{rel} = -1). Data are mean ± SEM, from six (GluA2, GluA2/γ2, and GluA2 3D/γ2) independent patch experiments.

(H) Conductance-voltage plots with 100 μM internal spermine for wildtype GluA2 (white circles), as well as GluA2/γ2 (blue triangles) and GluA2 3D/γ2 (black triangles) AMPAR-TARP fusion proteins. Conductance is normalized to the fitted maximal conductance (G_{max}), and corrected to account for the average conductance-voltage relationship in recordings without internal spermine (see Supplemental Experimental Procedures). Data are mean ± SEM, from six independent patch experiments for each condition (GluA2, GluA2/γ2, and GluA2 3D/γ2) with 100μM spermine.

Table S1 Data collection and refinement statistics (molecular replacement) for GluA2 K759M/T765K LBD crystal structures. Supplemental data associated with Figure 4.

Data set	GluA2 K759M/T765K zinc-form	GluA2 K759M/T765K lithium-form
**Data collection**		
Space group	*P* 2 2_1 2_1	*P* 1 2 1
Cell dimensions		
a, *b*, *c* (Å)	46.38, 110.52, 167.26	67.32, 47.56, 96.75
α, β, γ (°)	90, 90, 90	90, 95.65, 90
Resolution (Å)	2.90-92.2 (2.90-3.08) [a]	1.35-67.0 (1.35-1.39)
R_{meas}	0.225 (1.68)	0.074 (1.11)
$I / \sigma I$	4.5 (1.1)	10.92 (1.88)
CC(1/2)	98.8 (73.9)	99.7 (62.2)
Completeness (%)	100 (100)	94.7 (92.5)
Redundancy	5.0 (5.1)	3.5 (3.6)
**Refinement**		
Resolution (Å)	2.90	1.35
No. reflections	36,525	126,864
R_{work} / R_{free} [b]	0.243 / 0.283	0.161 / 0.178
No. atoms		
Protein	5873	4143
Ligands (Glu only)	30 (30)	52 (20)
Ions	5	2
Water	0	688
B-factors		
Protein	97.1	23.2
Ligands (Glu only)	89.7 (89.7)	33.9 (15.4)
Ions	119.4	11.2
R.m.s. deviations		
Bond lengths (Å)	0.006	0.011
Bond angles (°)	1.035	1.391

Data were collected from single crystals in each case.
[a] Values in parentheses are for highest-resolution shell.
[b] Tests sets for R_{free} contained 5% (zinc-form) or 2% (lithium-form) of total reflections.

Table S2 Time course of desensitization and deactivation for wildtype and mutant GluA2 receptors expressed alone, with auxiliary subunits, or as GluA2/γ2 fusion proteins. Supplemental data associated with Figures 6, 7, and 8.

	τ_{fast}	τ_{slow}	% fast	$\tau_{weighted}$	$\tau_{monoexponential}$	n
AMPAR subunits						
GluA2						
desensitization	7.9 ± 0.7	36.8 ± 8.3	94 ± 1	**9.2 ± 0.8**	**8.7 ± 0.71**	9
deactivation	0.61 ± 0.07	6.2 ± 0.9	96 ± 1	**0.76 ± 0.08**	**0.67 ± 0.07**	8
3D mutant series						
GluA2/γ2						
desensitization	22.4 ± 2.4	133.0 ± 17.3	79 ± 3	**45.7 ± 6.8**		11
deactivation	1.4 ± 0.3	13.8 ± 2.2	84 ± 3	**3.2 ± 0.4**		9
GluA2 3D/γ2						
desensitization	7.9 ± 0.9	32.3 ± 3.1	80 ± 3	**12.7 ± 1.2**		8
deactivation	0.83 ± 0.05	6.6 ± 1.2	93 ± 2	**1.1 ± 0.1**		8
GluA2 3D + γ2						
desensitization	5.8 ± 0.6	25.5 ± 3.7	76 ± 6	**9.5 ± 0.4**		7
deactivation	0.69 ± 0.05	16.4 ± 3.3	100 ± 0	**0.74 ± 0.04**	**0.67 ± 0.07**	7
AAA mutant series						
GluA2 + γ2						
desensitization	20.5 ± 3.6	86.1 ± 15.8	65 ± 7	**39.0 ± 4.4**		10
GluA2 + γ7						
desensitization	9.8 ± 1.0	58.7 ± 8.3	87 ± 1	**16.0 ± 1.5**		7
GluA2 AAA + γ2						
desensitization	2.5 ± 0.3	16.9 ± 1.4	73 ± 4	**6.6 ± 0.9**		8
GluA2 AAA + γ7						
desensitization	0.91 ± 0.10	27.0 ± 8.5	99 ± 0	**1.1 ± 0.1**	**0.92 ± 0.10**	6
GluA2 AAA/3D + γ2						
desensitization	1.1 ± 0.1	14.6 ± 2.3	89 ± 2	**2.4 ± 0.3**		7

GluA2 receptors were activated by long application (250 or 500 ms) or short (1 ms) applications of 10 mM glutamate to measure desensitization and deactivation kinetics, respectively. In the presence of auxiliary subunits, current decay associated with desensitization and deactivation was fit using bi-exponential functions to obtain the components τ_{fast} and τ_{slow}. Weighted time constants ($\tau_{weighted}$) were calculated based on the relative area fit by the fast and slow components. In cases where the τ_{fast} accounted for 94 % or more of the total area, the decay was instead fit by a monoexponential function, as reported as the value in the $\tau_{monoexponential}$ column. The number of patches for each condition (n) is indicated, and all values are mean ± SEM.

Table S3 Spermine affinities of GluA2 receptors. Supplemental data associated with Figure 7.

Fit parameters of GluA2 G/V relationships

Receptor		Mean	SEM	n
GluA2	Kd (0 mV) (μM)	2.7	0.4	
100 μM spermine	h (mV)	-17.3	0.6	6
	k (mV)	16.5	0.2	
0 μM spermine	G_0	1.09		
	V	53.2		
GluA2/γ2	Kd (0 mV) (μM)	16.5	1.3	
100 μM spermine	h (mV)	-14.0	1.0	6
	k (mV)	21.5	0.9	
0 μM spermine	G_0	1.14		
	V	82.4		
GluA2 3D/γ2	Kd (0 mV) (μM)	16.8	2.1	
100 μM spermine	h (mV)	-14.2	1.1	6
	k (mV)	25.2	0.6	
0 μM spermine	G_0	1.09		
	V	74.0		

Affinities were obtained using responses evoked by 250 or 500 ms applications of 10 mM L-Glu. Values for ‘h’ and ‘k’ indicate the voltage dependency. The number of patches for each condition (n) is indicated, and all values are mean ± SEM.

Movie S1A Lithium occupancy of the putative cation binding pocket of GluA2 K759M. Supplemental movie associated with Figure 1.

MD simulation of the GluA2 K759M LBD dimer shows that lithium (magenta) stably occupies the electronegative pocket throughout the simulation. Additional details regarding simulation parameters are described in the online methods section and quantification of the MD results are shown in Figure 1. The video encompasses 100 ns of the simulation.

Movie S1B Sodium does not occupy of the putative cation binding pocket of GluA2 K759M. Supplemental movie associated with Figure 1.

MD simulation of the GluA2 K759M LBD dimer shows that sodium (magenta) only rarely comes into contact with the electronegative pocket during the simulation. Additional details regarding simulation parameters are described in the online methods section and quantification of the MD results are shown in Figure 1. The video encompasses 100 ns of the simulation.

Movie S4A Rupture of T765K tether onto the cation pocket residues of GluA2 K759M/T765K. Supplemental movie associated with Figure 4.

MD simulation of the GluA2 K759M/T765K LBD dimer shows T765K initially maintaining contact with the electronegative residues that form the pocket, but then losing this interaction in the latter half of the simulation. Additional details regarding simulation parameters are described in the online methods section and quantification of the MD results are shown in Figure 4. The video encompasses 100 ns of the simulation.

Movie S4B Unstable tethering of T765K onto the cation pocket residues of GluA2 MKT. Supplemental movie associated with Figure 4.

MD simulation of the GluA2 MKT dimer shows T765K initially maintaining contact with the electronegative residues that form the pocket, but then having difficulty preserving this interaction in the latter half of the simulation. Additional details regarding simulation parameters are described in the online methods section and quantification of the MD results are shown in Figure 4. The video encompasses 100 ns of the simulation.

SUPPLEMENTAL EXPERIMENTAL PROCEDURES

DNA constructs

The GluA2/γ2 and GluK2/γ2 TARP fusion constructs were generated by large-insert site-directed mutagenesis (see (Geiser et al., 2001)). The γ2 coding sequence was amplified as part of a megaprimer, and then subsequently incorporated into plasmids encoding either the GluA2 or GluK2 iGluR subunit. The forward primer used to amplify the megaprimer corresponded to the C-terminal of the GluA2 or GluK2 (without its stop codon), a seven amino acid linker sequence ELGTRGS (Semenov et al., 2012), and the N-terminal of γ2. Likewise, the reverse primer corresponded to a region downstream of the iGluR subunit coding region and the C-terminal of γ2. The primer sequences used to generate the megaprimer for the GluA2/γ2 fusion protein were 5'- GGC ATC GAG AGT GTT AAA ATT GAA CTG GGT ACA CGA GGT TCT ATG GGG CTG TTT GAT CGA GGT G -3' (forward primer) and 5'- GTA ATT GAC AGC CTT GCC TTG CTC CTC ATT TCT CAT ACG GGC GTG GTC CG -3' (reverse primer), while for the GluK2/γ2 fusion protein they were 5'- CCA GGT AAA GAA ACT ATG GCA GAA CTG GGT ACA CGA GGT TCT ATG GGG CTG TTT GAT CGA GGT G -3' (forward primer) and 5'- CGA CAG TTT GTG CTT GGG TGA TTG GCC TCT TCT CAT ACG GGC GTG GTC CG -3' (reverse primer). All new constructs were screened by restriction digestion and confirmed by sequencing.

Electrophysiology

cDNA encoding AMPAR or KAR subunits and green fluorescent protein (eGFP) was typically co-transfected, but in some cases receptor subunit cDNA was present on plasmids also encoding eGFP behind an internal ribosomal entry site. For GluA1 and GluA2 AMPARs, the Q/R site was unedited, while the R/G site was unedited and edited, respectively. After transfection for 4 - 16

hours using the calcium phosphate precipitation method, cells were washed twice with divalent-containing PBS and maintained in fresh medium (MEM containing Glutamax and 10 % FBS), including 30 μM DNQX if auxiliary subunits were present. Electrophysiological recordings were performed 24 - 48 hours later. The osmotic pressure of recording solutions was adjusted to 300 mOsm using sucrose, while the pH was typically adjusted to 7.4 with alkali hydroxide solutions. For experiments involving spermine in the patch pipette the internal solution contained (in mM): 120 NaCl, 10 NaF, 5 HEPES, 5 Na_4BAPTA, and 0.5 $CaCl_2$ with 100 μM spermine added on the day of experiments. In the case of recordings conducted without external NaCl, the solution contained 100 μM of $CaCl_2$ and $MgCl_2$ to improve patch stability, sucrose to maintain the osmotic pressure at 300 mOsm, and 5 mM Tris to buffer pH. The pH was further adjusted to 7.4 using 10 N HCl. In the case of single-channel recordings, internal solution typically contained (in mM): 135 CsF, 33 CsOH, 10 HEPES, 11 EGTA, 1 $CaCl_2$, and 2 $MgCl_2$.

All experiments were performed on excised membrane patches in the outside-out configuration. Recording pipettes were composed of borosilicate glass (3-5 MΩ, King Precision Glass) coated with dental wax, or quartz glass (3-15 MΩ, King Precision Glass) coated with Sylgard (Dow Corning) to obtain recordings of single channels or for stable recordings in external solution without NaCl. The reference electrode was connected to the bath via an agar bridge of 3M KCl. The holding potential during recordings was -60 mV (unless otherwise stated). Series resistances (3-15 MΩ) were routinely compensated by 95 %. For single-channel recordings, the headstage was set to the capacitive feedback recording mode. All recordings were performed using an Axopatch 200B amplifier (Molecular Devices). Current records were low-pass filtered by an 8-pole Bessel filter at 10 kHz and sampled at 25-100 kHz for population responses or 100 kHz for

single-channel responses. Data were acquired using pClamp9 software (Molecular Devices) and illustrated using Origin 7 (OriginLab).

Analysis of Electrophysiological Data

Electrophysiological data containing population and single-channel responses were analyzed using Clampfit 9.0 (Molecular Devices) and Signal 5.0 (Cambridge Electronic Design), respectively. Current decay rates were fit using 1st or 2nd order exponential functions: $y = A_i *\exp(-x/t_i)$, with the latter used when auxiliary subunits were present (see Table S2). Single-channel data were processed as described previously (Dawe et al., 2013). In brief, digital low-pass filtering at 3 kHz was performed prior to time-course fitting, which resulted in root mean square baseline noise values that averaged 0.22 ± 0.02 pA (n = 4) and 0.18 ± 0.01 pA (n = 4) for wildtype GluA2 and MKT mutant receptors, respectively. These noise values corresponded to approximately fifty percent of the smallest conductance level. Idealized record response amplitudes were fit with Gaussian functions, whose peaks reflect discrete conductance levels: $y = \sum_{i=1...n} (A_i/(w_i*\mathrm{sqrt}(\pi/2)))*\exp(-2*((x-xc_i)/w_i)^2)$ where A = area, xc = center of the peak, w = error associated with xc. Open probability was calculated for each patch containing GluA2 MKT as the percentage of open time in the idealized record.

Fitting of Conductance Voltage Relationships

Agonist-evoked membrane conductance (G) was calculated using the equation: $G = I / (V – V_{rev})$, where I is the current at V holding potential, and V_{rev} is the reversal potential. Conductance-voltage (G/V) relationships were fit using Origin 7 (OriginLab) with two different equations (Bowie et al., 1998). For recordings without internal polyamines, $y = (1 + (G_0 – 1)*\exp(x/V))$ was used, where G_0 is the minimal conductance and V is the holding potential. For recordings with internal polyamines, $y = G_{max} / (1 + [PA]/(g*\exp(x/h) + L*\exp(x/k)))$ was used,

where G_{max} is the maximal conductance and [PA] is the concentration of polyamine (in μM), such that the polyamine dissociation constant, K_d = g*exp(V/h) + L*exp(V/k) (see (Bowie et al., 1998)). For each receptor studied, the K_d(0 mV) and the voltage-dependent rates h an k are reported (Table S3). Conductance-voltage data from patch recordings with internal polyamines were corrected based on the average conductance profile of the same receptor without polyamines. In some cases, residual polyamine block was detected during outside-out patch recordings, despite the presence of 10 mM ATP to chelate polyamines in the patch pipette. To eliminate this block during control experiments, a train of L-Glu pulses at -80 mV were delivered prior to the test pulse, as described previously (Rozov et al., 1998).

Molecular dynamics simulations

The L-Glu-bound GluA2 (flip, R/G unedited) LBD structure (PDB # 2UXA (Greger et al., 2006); resolution 2.38 Å) was obtained from the Research Collaboratory for Structural Bioinformatics (RCSB) protein data bank (Berman et al., 2000). For 2UXA, chains A and C were used, and for the K759M/T765K mutant structure, chains A and B were used. Zinc ions were removed in both cases before simulation setup, and for all simulations based on the 2UXA structure, the R764G mutation was introduced. Missing atoms were added in PyMOL (The PyMOL Molecular Graphics System, Version 1.4, Schrödinger) and missing residues were added using Modeller Version 9.12 (Sali and Blundell, 1993). The LBD dimer was solvated in a cubic water box with dimensions $(100\ \text{Å})^3$ using the TIP3P water model (Jorgensen et al., 1983), and subsequently the system was neutralized and 150 mM NaCl or LiCl was added. Mutations were imposed manually prior to simulation setup, either by editing/deleting atoms in the pdb-file or by using the mutate function of PyMOL (The PyMOL Molecular Graphics System, Version 1.4, Schrödinger) and adjusting the side chain rotamer.

For MD simulations, the systems were first energy minimized until the maximum force on an atom was less than 100 kJ/mol/nm. Following energy minimization, a 200 ps restrained simulation with position restraints on protein heavy-atoms with a force constant of 1000 kJ mol^{-1} nm^{-2} was performed in the NVT ensemble with a temperature of 300 K maintained by a Berendsen thermostat (Berendsen et al., 1984). Periodic boundary conditions were employed and van der Waals interactions were cut off at 10 Å. Long-range electrostatics were accounted for by the Particle-Mesh Ewald method (Essmann et al., 1995). All bonds were treated as constraints using the LINCS algorithm (Hess, 2008), allowing a time step of 2 fs. Subsequently, another 200 ps restrained simulation was performed as above but in the NPT ensemble at a pressure of 1 bar, maintained by a Berendsen barostat (Berendsen et al., 1984). Following this, 100 ns of production run were performed.

X-ray crystallography.

During protein purification, cell pellets were incubated in high sucrose buffer (20 % (w/v) sucrose, 25 mM HEPES pH 8.0, 5 mM EDTA, 0.25 mg/ml lysozyme) for 45 min at room temperature, spun (2,000x g, 30 min, 4°C) and the pellets frozen at -80°C. These were thawed into 25 mM HEPES pH 7.5, 150 mM NaCl, 5mM L-Glu, 0.25 U/ml benzonase (Sigma), incubated (30 min at room temperature) and spun (18,500x g, 30 min, 4°C). Protein-containing supernatant was then purified (see main Experimental Procedures).

Crystals were grown in hanging-drops by mixing purified protein (5-10 mg/ml in 25mM HEPES, 150 mM NaCl, 5 mM L-Glu) in a 1:1 ratio with well solution containing either lithium (20-22 % PEG 4,000, 200 mM lithium sulfate, 100 mM acetate pH 5.0; grown at 6°C) or zinc ions (12-15 % PEG 8,000, 200 mM zinc acetate, 100 mM MES pH 6.0; grown at 23°C). Crystals grew in 1-3 weeks and were cryo-protected by briefly soaking in well solution containing 20-

22.5 % glycerol prior to plunge-freezing in liquid N_2. Diffraction limits were chosen based on a combination of $I / \sigma I > 1$, CC(1/2) > 0.5, and completeness in the outer shell > 90%. Molecular replacement in PHASER used the 2UXA GluA2 (flip) LBD structure as a model, modified with residues K759 and T765 truncated to Ala. Ramachandran statistics were 99.2/0.8/0.0 (% favored/allowed/outlier) for the zinc form and 99.0/1.0/0.0 for the lithium form.

SUPPLEMENTAL REFERENCES

Berman, H.M., Westbrook, J., Feng, Z., Gilliland, G., Bhat, T.N., Weissig, H., Shindyalov, I.N., and Bourne, P.E. (2000). The Protein Data Bank. Nucleic Acids Res 28, 235-242.

Bowie, D., Lange, G.D., and Mayer, M.L. (1998). Activity-dependent modulation of glutamate receptors by polyamines. J Neurosci 18, 8175-8185.

Essmann, U., Perera, L., Berkowitz, M.L., Darden, T., Lee, H., and Pedersen, L.G. (1995). A Smooth Particle Mesh Ewald Method. J Chem Phys 103, 8577-8593.

Geiser, M., Cebe, R., Drewello, D., and Schmitz, R. (2001). Integration of PCR fragments at any specific site within cloning vectors without the use of restriction enzymes and DNA ligase. BioTechniques 31, 88-90, 92.

Hess, B. (2008). P-LINCS: A parallel linear constraint solver for molecular simulation. J Chem Theory Comput 4, 116-122.

Jorgensen, W.L., Chandrasekhar, J., Madura, J.D., Impey, R.W., and Klein, M.L. (1983). Comparison of Simple Potential Functions for Simulating Liquid Water. J Chem Phys 79, 926-935.

Lomeli, H., Mosbacher, J., Melcher, T., Hoger, T., Geiger, J.R., Kuner, T., Monyer, H., Higuchi, M., Bach, A., and Seeburg, P.H. (1994). Control of kinetic properties of AMPA receptor channels by nuclear RNA editing. Science 266, 1709-1713.

Nayeem, N., Zhang, Y., Schweppe, D.K., Madden, D.R., and Green, T. (2009). A nondesensitizing kainate receptor point mutant. Mol Pharmacol 76, 534-542.

Rozov, A., Zilberter, Y., Wollmuth, L.P., and Burnashev, N. (1998). Facilitation of currents through rat Ca2+-permeable AMPA receptor channels by activity-dependent relief from polyamine block. J Physiol 511 (Pt 2), 361-377.

Sali, A., and Blundell, T.L. (1993). Comparative protein modelling by satisfaction of spatial restraints. J Mol Biol 234, 779-815.

Semenov, A., Moykkynen, T., Coleman, S.K., Korpi, E.R., and Keinanen, K. (2012). Autoinactivation of the stargazin-AMPA receptor complex: subunit-dependency and independence from physical dissociation. PLOS ONE 7, e49282.
